# The intertemporal evolution of agriculture and labor over a rapid structural transformation: Lessons from Vietnam^☆^

**DOI:** 10.1016/j.foodpol.2020.101913

**Published:** 2020-07

**Authors:** Yanyan Liu, Christopher B. Barrett, Trinh Pham, William Violette

**Affiliations:** a International Food Policy Research Institute, United States; b Dyson School of Applied Economics and Management, Cornell University, United States; c Dyson School of Applied Economics and Management, Cornell University, United States; d Federal Trade Commission, United States

**Keywords:** Vietnam, Structural transformation, Rural labor market, Inverse farm size and productivity relationship

## Abstract

We combine nationally representative household and labor force survey data from 1992 to 2016 to provide a detailed description of rural labor market evolution and how it relates to the structural transformation of rural Vietnam, especially within the agricultural sector. Our study adds to the emerging literature on structural transformation in low-income countries using micro-level data and helps to answer several policy-related questions. We find limited employment creation potential of agriculture, especially for youth. Rural-urban real wage convergence has gone hand-in-hand with increased diversification of the rural economy into the non-farm sector nationwide and rapid advances in educational attainment in all sectors’ and regions’ workforce. Minimum wage laws seem to have played no significant role in increasing agricultural wages. This enhanced integration also manifests in steady attenuation of the longstanding inverse farm size-yield relationship. Farming has remained securely household-based and the family farmland distribution has remained largely unchanged. Small farm sizes have not obstructed mechanization nor the uptake of labor-saving pesticides, consistent with factor substitution induced by rising real wage rates. As rural households rely more heavily on the labor market, human capital accumulation (rather than land endowments) have become the key correlate of improvements in rural household well-being.

## Highlights

• The rural economy has increasingly diversified into the non-farm sector nationwide.
• Real wages have increased rapidly and the inter-sectoral wage gap has widened.
• Small farm sizes have not obstructed mechanization nor the uptake of labor-saving pesticides.
• The longstanding inverse farm size-yield relationship has steadily attenuated over time.
• Human capital accumulation is the key correlate of improvements in rural household well-being.

## 1 Introduction

Over the past three decades, Vietnam has undergone one of the most rapid structural transformations of any low-income agrarian nation in history. Since the Doi Moi reforms initiated in late 1986 with the objective of creating a robust, socialist-oriented, market economy out of what had been a fully centrally-planned one, Vietnam has consistently experienced well-above-average real GDP growth of 4–8 percent annually.^1^ From 1992 – the earliest year for which nationally-representative survey data are available – to 2016, per capita real gross national income (GNI) more than tripled, turning Vietnam from one of the world’s poorest countries, at less than US $500/year (in constant 2010 dollars) to roughly $1700, while the share of GDP in agriculture fell from 34% to 16% and the share of the workforce employed in agriculture fell from 68% to 42% (World Bank 2019). Put differently, in 1992 Vietnam’s income level and economic structure was strikingly similar to present day Liberia. Today it is a solidly (lower) middle-income country enjoying comparatively rapid economic growth and poverty reduction. Starting as one of the poorest, most agriculture-intensive economies with still-above-average population growth, Vietnam provides a valuable case study for understanding the structural transformation process among low-income countries and its implications for agriculture and labor markets. Since the global poor disproportionately reside in rural areas and work (at least part-time) in agriculture, understanding this process is essential to developing viable poverty reduction strategies in today’s low-income agrarian economies.

Our study is motivated by several current policy concerns in developing countries. One, does structural transformation lead to regional specialization, with rural areas concentrating in farming and losing workers who leave for cities? Do we see rapid transition into the non-farm sector within rural areas? To what extent does an increasingly productive farming sector absorb young workers in low-income countries (Losch, 2016, Mueller and Thurlow, 2019)? Two, do real wages for farmworkers converge to those earned by non-farm workers, and do rural wage rates converge to urban rates? Do minimum wage laws enacted primarily for urban non-farm workers seem to bind in the agricultural sector (Belman and Wolfson, 2015, Bhorat et al., 2017)? Three, does structural transformation lead to family farm consolidation, mechanization, and the displacement of workers as farms grow larger and more mechanized (Mrema et al., 2008, OECD, 2016)? Does the commonly observed inverse farm size–productivity relationship in developing countries attenuate, suggesting more competitive and integrated rural factor and output markets and less rationale for agricultural policies favoring smaller farms? Four, is structural transformation associated with rapid increases in well-being for households that remain in rural areas (Barrett et al., 2017, Christiaensen and Martin, 2018)?

This paper offers descriptive evidence on the structural transformation process that Vietnam has undergone. We combine nationally representative household data (Vietnam Household Living Standards Surveys, VHLSS) from 1992 to 2016 and labor force survey (LFS) data from 2007 to 2016 in order to provide a detailed description of rural labor market evolution and how it relates to the transformation of Vietnamese agriculture. This paper thereby adds to the emerging literature on structural transformation in low-income countries using micro-level data. Most studies on structural transformation rely on macro-level data (Timmer, 2002, Timmer, 2009; Rodrik, 2013, Dercon and Gollin, 2014, Gollin, 2014, Gollin et al., 2016, Rodrik, 2016, Diao et al., 2017), while micro-level data shed light on transformation within one country and unmask potential heterogeneity in the growth process (McCullough, 2016, McCullough, 2017).^2^ To our knowledge, our study covers a longer period (24 years) than do prior studies using microdata.

For any of several reasons, the Vietnamese case might not provide an apt lens through which to view today’s low-income agrarian economies. Vietnam has retained an extraordinarily strong state while shifting from a centrally planned to a market economy. Its proximity to the east Asian boom economies of China, Korea, and Taiwan, along with normalization of relations with the United States, has enabled buoyant export growth,^3^ which is perhaps less accessible to other low-income agrarian nations. The country has a high population density, but rapidly slowing population growth, leading to an aging population. The central government invested heavily in education and health, leading to educational performance and health indicators more characteristic of upper-middle- and high-income countries. Nonetheless, the Vietnamese experience can be instructive for today’s low-income agrarian economies, in part because its experience contrasts with some common narratives of what might be inevitable as such economies undergo structural transformation.

We have four main findings. First, Vietnamese households have diversified out of agriculture, manifest not only in decreasing shares of farming households, but also in a decline in the agricultural labor force within farming households, and in the agricultural income share of rural households.^4^ We also observe uneven structural transformation across regions, with sharper reduction in the share of agricultural labor forces in more urbanized Red River Delta and Southeast regions. Second, real wages have increased rapidly in both the farm and nonfarm sectors, seemingly driven by rapid advances in educational attainment and not by changes in statutory minimum wage rates. The nonfarm sector has seen significantly higher real wage growth than the farm sector and the inter-sectoral wage gap has widened, although this may reflect selection on human capital (Coxhead et al., 2016). Increasing employment in higher-productivity nonfarm sectors points to a successful structural transformation, which contributes to the overall economic growth (McMillan and Rodrik 2011). And real wage growth faster than overall GDP expansion indicates an inclusive growth process benefitting workers disproportionately. Third, the rapid structural transformation does not lead to family farm consolidation.^5^ Family farm size remains small and the land distribution changed remarkably little over two-plus decades of rapid rural transformation. Nevertheless, mechanization – mainly through rental markets – and the use of labor-saving inputs like pesticides has grown steadily, likely driven by rising labor costs and farmers’ improved access to finance as farm productivity grew. Rice yields continued increasing. In line with more efficient factor and output markets, over time the commonly observed inverse farm size–productivity relationship has attenuated. Fourth, as rural households rely more heavily on the labor market rather than agriculture, human capital accumulation plays an increasingly more important role in household well-being. In contrast, land endowment becomes less strongly associated with per capita consumption expenditures over time, further underscoring the transition from an agrarian economy where sector-specific assets such as land are the main determinants of income to a more modern economy based more on the returns to accumulated human capital.

McCaig, 2017, Tarp, 2017 also describe structural transformation in Vietnam. Using the 1990s-2000s nationally representative household survey and population census data, McCaig and Pavcnik (2017) document that, as the nonfarm sectors provided more job opportunities, people moved out of farming, driving up agricultural real wages and shrinking the rural–urban wage gap between agricultural and nonagricultural sectors.^6^ Tarp (2017) also describes the rural transformation, relying on the 2006–2014 Viet Nam Access to Resources Household Survey (VARHS) data. VARHS focuses on rural areas and is not nationally representative, however.

Our study builds on these two studies in at least four aspects. First, we use a longer duration of nationally representative data to document the evolution of rapid structural transformation over 24 years in Vietnam. Second, we provide a more detailed description of rural, and especially agricultural transformation. Third, we complement the nationally representative household survey data with LFS data to provide a more detailed description of rural labor markets and a cross-check on the household survey data. Fourth, we show important, policy-relevant findings beyond those from the existing studies, including the increasing role of human capital (and declining role of land holdings) in determining welfare of rural households, the attenuation of farm size-productivity relationship over time, as well as increased role of machinery in farming.

The paper proceeds as follows. Section 2 describes the data. Section 3 describes the evolution of rural and agricultural labor markets. Section 4 describes the evolution of the agricultural sector. Section 5 explores the evolution of well-being among rural households. Section 6 concludes.

## 2 Data

The data that we use come from two nationally representative data sets, the ten-round VHLSS from 1992 to 2016 and the six-round LFS from 2007 to 2016. Such rich, nationally representative descriptive analysis is uncommon during an extended period of rapid growth.

### 2.1 Vietnam household Living Standards surveys (VHLSSs)

This study uses data from the 1992 and 1998 Vietnam Living Standards Surveys (VLSS) and from the 2002–2016 biennial Vietnam Household Living Standards Surveys (VHLSS) (General Statistics Office of Vietnam, GSOV). For simplicity, we use VHLSS to refer to both the VLSS and VHLSS throughout. The VHLSSs are representative at national, regional, urban, rural, and provincial levels. Therefore, the subnational breakdowns that we report are all statistically representative of those populations. The VHLSSs use similar survey instruments throughout the 1992–2016 period, including a household questionnaire and a commune questionnaire. The 1992 VHLSS sampling frame was the 1989 census. The VHLSS 1998 intended to interview all households from the VHLSS 1992. The 2002 to 2008 VHLSSs were sampled based on the 1999 census and the 2010–2016 rounds were sampled based on 2009 census. In each 2002–2008 VHLSS round and in the 2012–2016 VHLSS rounds, half of the communes surveyed in the previous round were randomly selected and the sample households in these selected communes were re-interviewed, and another group of households was added to the sample to construct a rotating panel.^7^

For some analyses, we merge the data from household and commune surveys to construct five rural household panels: VHLSS 1992/1998, VHLSS 2002/2004, VHLSS 2006/2008, VHLSS 2010/2012, and VHLSS 2014/2016. Appendix Table A1 summarizes the number of households and number of communes in each panel.^8^ The household survey data provide information on household demographics, rice output by type of rice, rice planting area, landholdings, labor hiring, agricultural inputs, consumption expenditure, and income from different sources. The commune survey provides information on local agricultural wage by gender and by task (land preparation, planting, tendering, and harvesting). We generate median wage rates by gender, combining across all tasks for each commune and each panel round. To calculate the real wage and consumption expenditure, we deflate local nominal wage rates and nominal expenditure by the national consumer price index (CPI), which captures intertemporal inflation, and by the CPI at the region-rural/urban level. Sampling weights are applied throughout.

### 2.2 Labor force survey (LFS)

The Labor Force Survey (LFS) has been conducted by the Government Statistics Office of Vietnam with technical support from the International Labor Organization in 2007, 2009, and annually since 2010. LFS is representative at the national, urban, rural, and regional level.^9^ The LFS surveys consist of household and employment information. The household part provides brief demographic characteristics, such as relationship to the household head, gender and year of birth of all members of each household. The employment part is focused on employment aspects, such as highest general education and/or technical education qualification, economic activities, employment status, occupation, industry in which the person is working, hours worked, and income/wage of individuals aged 15 and above. Individuals are categorized into five sub-groups: fully-employed, under-employed, un-employed, discouraged, and other; of which the first three are considered workforce, whereas the latter two are considered out of the workforce. Appendix Table A2 summarizes the number of individuals aged 15 and above surveyed in each round.

## 3 Evolution of rural and agricultural labor markets

### 3.1 Evolution of agricultural and non-farm employment patterns

Table 1 reports the share and number of households working in agriculture (including forestry and aquaculture) in each survey round from 1992 to 2016, for all of Vietnam and by region, based on VHLSS data.^10^ The share of households engaged in agriculture fell from 83.5% in 1992 to 62.9% in 2016 (a slight uptick from the 61.6% low in 2014). Subnational results show large disparities in the evolution of employment in agriculture. As the most economically developed region – home to the largest municipality (Ho Chi Minh City) – the Southeast region had the lowest share of agricultural households over this period. In 2016, only 30.7% of households were involved in agriculture, down from 53.3% in 1992 in this relatively urban region. The Red River Delta region, home to Hanoi (Vietnam's capital and second largest city by population), experienced the fastest transition out of agriculture among all regions, as the share of households involved in agriculture decreased from 83.8% in 1992 to 56.3% in 2016. We also see the emergence of regional differentiation over this period. Omitting the Southeast, which was an outlier in 1992, the inter-region range of the share of agricultural households expanded considerably, from 0.84 to 0.96 in 1992 to 0.56–0.83 in 2016. Even in 2016, the most agrarian, rural provinces – the Central Highlands, North and South-Central Coast, and North East and North West – still had three-quarters or more of households engaged in farm production.

**Table 1:** Share and Number of Households Involved in Agriculture, 1992–2016.

	1992	1998	2002	2004	2006	2008	2010	2012	2014	2016
Panel A: Share of households involved in agriculture										
Rural	0.950	0.949	0.907	0.895	0.876	0.859	0.820	0.819	0.809	0.810
Total	0.835	0.786	0.758	0.744	0.705	0.683	0.637	0.643	0.616	0.629
By region										
Central Highlands	0.961	0.981	0.894	0.872	0.850	0.839	0.805	0.794	0.805	0.814
Mekong River Delta	0.844	0.801	0.787	0.773	0.733	0.740	0.675	0.695	0.664	0.666
North and South-Central Coast	0.955	0.909	0.872	0.873	0.836	0.802	0.798	0.805	0.811	0.826
North East and North West	0.875	0.855	0.819	0.809	0.773	0.752	0.746	0.747	0.712	0.749
Red River Delta	0.838	0.796	0.777	0.765	0.717	0.694	0.651	0.636	0.605	0.563
South East	0.533	0.480	0.439	0.415	0.391	0.354	0.297	0.316	0.296	0.307
Panel B: Number of households involved in agriculture										
Rural		11.628	11.883	11.739	12.465	12.968	12.692	13.310	12.950	13.907
Total		12.671	13.102	13.042	13.843	14.318	14.197	14.922	14.915	15.775
By region										
Central Highlands		0.357	0.784	0.741	0.864	0.925	0.970	0.999	1.093	1.246
Mekong River Delta		2.619	2.795	2.730	2.905	3.098	2.826	3.038	3.063	2.988
North and South-Central Coast		2.514	2.094	2.162	2.317	2.331	2.479	2.668	2.650	3.127
North East and North West		3.072	3.069	3.069	3.233	3.307	3.302	3.509	3.358	3.820
Red River Delta		2.930	3.263	3.244	3.334	3.464	3.390	3.344	3.360	3.244
South East		1.180	1.096	1.097	1.191	1.194	1.229	1.361	1.391	1.350

Note: The sample includes household from 10-round VHLSS from 1992 to 2016.

Consistent with household-level results from VHLSS, individual-level LFS data reveal similar patterns (Appendix Table A3). The share of individual workers employed in agriculture declined from 48.4% to 39.4% from 2007 to 2016. The fact that these shares are far less than the proportion of agricultural households signals that even agricultural households have long diversified their earnings portfolios across sectors (on which, more below), as is true in sub-Saharan African low-income agrarian nations as well (Barrett et al. 2001). Such a pattern was also observed in today’s high-income countries during their structural transformations.

This increasing diversification into non-farm employment is perhaps best seen by looking at individual household members’ employment in farming (as farmers or farm workers) within farming households. Panel A of Table 2 reports the mean share of farming household members who are full-time farmers or farm workers, defined as 35 h or more per week spent working in agriculture. On average, full-time farmers only accounted for 16.7% of total household members in 2002, declining to 9.0% in 2016. Even among the members engaged in farming, full-time farmers only accounted for 31.2% in 2012 and 16.0% in 2016. Farming is clearly a part-time activity for most people employed in it

**Table 2:** Members’ engagement in farming within farming households.

	1992	1998	2002	2004	2006	2008	2010	2012	2014	2016
Panel A: Mean share of farming household members who are full-time farmers/farm workers										
Full-time/Total household members	–	–	0.167	0.128	0.125	0.125	0.096	0.100	0.097	0.090
Full-time/Total self-employed farm members	–	–	0.329	0.243	0.241	0.232	0.181	0.184	0.185	0.172
Full-time/Total (self-employed/waged) farm members	–	–	0.312	0.233	0.232	0.223	0.170	0.171	0.173	0.160
Panel B: Mean share of farming household members involved in nonfarm activities										
Self-employed Nonfarm members	0.132	0.131	0.119	0.127	0.120	0.111	0.107	0.100	0.101	0.103
Waged Nonfarm members	0.046	0.055	0.094	0.117	0.126	0.130	0.135	0.142	0.156	0.166
(Self-employed/Waged) Nonfarm members	0.174	0.183	0.209	0.238	0.242	0.237	0.239	0.240	0.254	0.265
Panel C: % farming households involved in nonfarm activities										
Self-employed nonfarm activities	0.376	0.366	0.332	0.348	0.328	0.303	0.282	0.263	0.269	0.269
Waged nonfarm activities	0.168	0.196	0.305	0.359	0.374	0.385	0.384	0.395	0.422	0.444

Notes: The sample includes households from VHLSS 1992–2016. Full-time refer to those who work at least 35 h per week.

Panel B of Table 2 reports the mean share of farming household members involved in nonfarm activities (self-employed or as wage/salaried laborers). Rural households have steadily diversified out of agriculture, especially into wage labor in the non-farm sector. In 1992, only 4.6% of farming household members had non-farm work paying a wage; by 2016, that number had climbed to 16.6%, surpassing the proportion employed in farming. Much of this expansion came at the extensive margin, as farming households began to place family members into wage non-farm employment for the first time. The share of farming households with a member earning non-farm wages increased from 16.8% in 1992 to 44.4% by 2016. Furthermore, the rise of wage non-farm employment by farming household members also eclipsed non-farm self-employment over this period. In 1992, 37.6% of farming households were also engaged in non-farm self-employment, but that share declined steadily over time to 26.9% in 2014–2016. Combined, the share of farming household members employed in (wage or self-employed) non-farm activities grew from 17.4% to 26.5% over the 1992–2016 period.

**Fig. 1:**
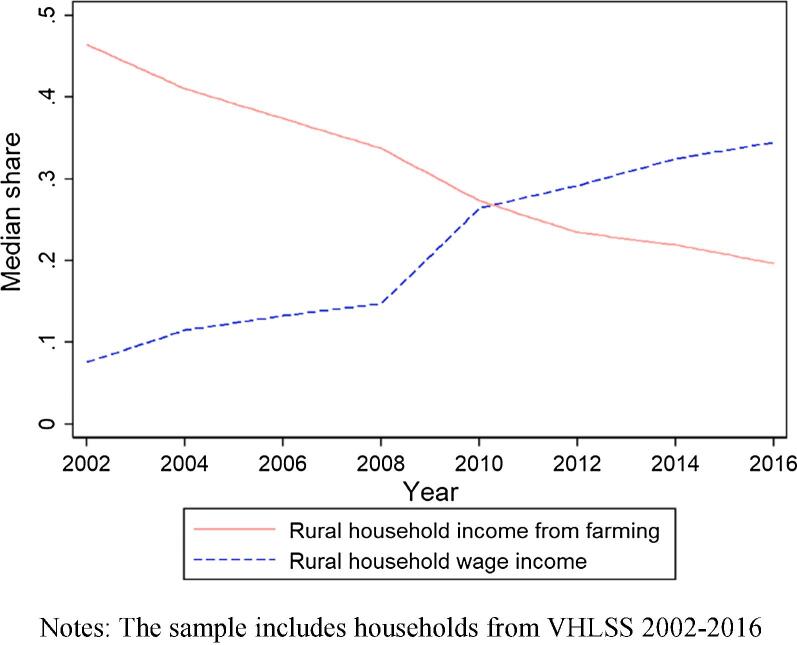
Median share of income of rural households from farming and from wage earnings. Notes: The sample includes households from VHLSS 2002–2016.

Another way to grasp the sharp, nationwide transition of rural households towards non-farm, and especially wage labor, is through the shares of total household income arising from agricultural versus wage earnings. As shown in Fig. 1, from 2002 to 2016, the median share of rural households’ income from agriculture declined from 0.465 to 0.197; and the median share of wage income increased sharply from 0.076 to 0.345. Since 2010, wage income has represented a larger share of median rural household incomes than agricultural earnings do. This figure perhaps best represents the dramatic structural transformation of the rural Vietnamese economy over this period, as agriculture has become less important as an employer and as a source of income for households even as its productivity has increased sharply and the use of modern inputs that boost labor productivity – e.g., fertilizers, improved seeds, machinery, pesticides – has increased rapidly (see section 4). Table A4 summarizes the evolution of the median shares of agricultural income and wage income, by region. All regions share the common trend that the income share from farming continuously reduces while the share from wages continuously increases. In the South East and the Red River regions, a median rural household only had 2.3% and 7.8% income from farming in 2016, respectively. Rural households’ lower dependence on farming is most pronounced in the Red River region, which saw a sharp reduction of median income share from farming, from 41.1% to 7.8%, during the merely 14-year period. This is consistent with the rapid urbanization surrounding the metropolitan area of Hanoi. This resembles patterns in high income countries, where even within the farm sector, most households earn more net income from wages than from agriculture.

Vietnamese agriculture is traditionally dominated by farmers who cultivate their own land. This pattern remained largely unchanged during the structural transformation. Among the population employed in agriculture, only 5.1% were hired workers in 1992. That share increased very slowly, to just 8.0% by 2016.^11^ Farming has remained concentrated among households with access to land who employ predominantly family labor on the farm. The corollary to this is that Vietnam has not seen the emergence of a farmworker class.^12^

Table 3 reports the agricultural labor force composition by age, gender, and education levels from 2007 to 2016. Panel A shows that the shares of the agricultural labor force in age groups below 50 years old (15–20, 20–30, 30–40, 40–50) all declined over this period while older age groups (50–60, 60–70, and 70 + ) accounted for increasing shares of the agricultural labor force. This observation may partly reflect the aging of the overall labor force in Vietnam. However, the aging in agricultural labor force is more severe than in the overall labor force. The share of labor force younger than 50 years old fell from 75 to 60 percentage points (a 20% reduction) for agriculture only, in comparison with a 10% reduction (from 81 to 73 percentage points) for the overall labor force. The aging of the national and agricultural labor force is in line with broader demographic patterns within the Vietnamese population, as shown in Appendix Figure A1 which plots population pyramids by age and gender, for all Vietnam and rural only, from the 1989, 1999, and 2009 census. A sharp reduction in fertility rates manifests in a rapid demographic transformation that does not differ meaningfully between rural and urban areas.^13^

**Table 3:** Labor force composition by age, gender and education, 2007–2016.

	Agricultural labor force					Total labor force				
	2007	2011	2012	2014	2016	2007	2011	2012	2014	2016
Panel A: Age										
15–20	8.03	7.23	5.87	5.91	4.78	6.78	5.97	5.13	4.72	3.76
20–30	19.22	17.95	17.25	15.90	14.25	23.42	23.34	22.16	21.07	20.78
30–40	22.93	21.19	20.52	19.55	18.35	25.80	25.01	24.62	24.31	24.92
40–50	24.95	23.69	24.21	23.07	22.58	24.64	23.43	24.31	23.65	23.41
50–60	15.87	18.78	20.21	21.95	24.19	13.48	15.44	16.48	18.02	18.33
60–70	6.60	7.93	8.61	10.15	12.21	4.33	4.97	5.43	6.33	7.01
70 and above	2.40	3.24	3.33	3.47	3.64	1.55	1.84	1.88	1.90	1.81
Panel B: Education level										
None	36.40	25.03	24.76	24.06	21.61	26.29	19.68	19.97	19.65	13.22
Primary school	48.72	29.60	30.25	29.79	29.51	43.36	24.42	24.51	23.61	22.95
Lower secondary school	9.78	35.57	34.70	34.87	35.84	14.41	31.53	30.72	30.23	31.44
Upper secondary school	2.94	8.08	8.38	8.84	9.85	3.55	12.46	12.38	12.58	16.04
College and above	2.16	1.72	1.91	2.45	3.20	12.39	11.90	12.42	13.93	16.36
Panel C: Gender										
Male	0.465	0.466	0.467	0.472	0.470	0.508	0.513	0.515	0.514	0.515

Notes: The sample includes individuals from LFS 2007–2016.

Panel B of Table 3 indicates that the education level (i.e., highest level of education completed) of the agricultural labor force steadily increased during this period, consistent with improvements in education for the overall labor force in Vietnam. From 2007 to 2016, the share of the agricultural labor force that had never attended school fell from 36% to 22%, and the share of workers with lower secondary education or above dramatically increased from 15% to 49%. This reflects the rapid rise in education nationwide. Moreover, with merely 10% possessing upper secondary education and 3% with college education or above, the agricultural labor force still had much lower educational levels than the overall labor force, within which 16% of workers had completed upper secondary school and another 16% college or above in 2016. The government’s massive investments in education were clearly translating into a better educated workforce, with those gains accruing in all sectors, but disproportionately in the non-farm sectors. This is not surprising given that younger and better-educated populations have higher propensity to migrate from agriculture to nonfarm sectors (Hicks et al. 2017; Young, 2013). Migration also contributes to aging of agricultural labor force which is commonly observed globally.

The gender composition of the agricultural labor force was virtually unchanged during this period (Table 3 Panel C). Throughout the 2007–2016 period, male labor accounted for 46–47% of the overall agricultural labor force. The nation’s total labor force, by contrast, was slightly majority male, at 51–52% throughout this period. Women’s labor force participation rates, including in agriculture, are thus roughly comparable to men’s in Vietnam. This is remarkable, given that International Labor Organization (ILO, 2018) estimates the global gap in women’s labor force participation at 27 percent. Indeed, Vietnam has maintained a 70% women’s labor force participation rate for two decades, which is not only higher than any other developing countries in Asia but is unsurpassed by many advanced economies (Banerji et al. 2018). Despite gender equality in labor force, which is partially attributed to gender parity in education attainment (Banerji et al. 2018), women still suffer wage penalties on average (more discussion in Section 3.2).

### 3.2 Growth in real agricultural wages

The VHLSS and LFS surveys provide complementary measures of real agricultural wages over the structural transformation. Real wage measures from the VHLSS come from the commune module in which commune leaders approximate the average daily payment for farm work in their communes. By contrast, individual workers in both agricultural and non-agricultural sectors report their wages directly in the LFS. While direct reports from the LFS may include less measurement error than commune leader reports of local averages from the VHLSS, the LFS may also produce biased estimates by underreporting part-time, seasonal, or migrant labor. Comparing the evolution of real wages in both surveys may help to identify changes in real agricultural wages that are robust to sampling designs and different sources of non-classical measurement error.

**Fig. 2:**
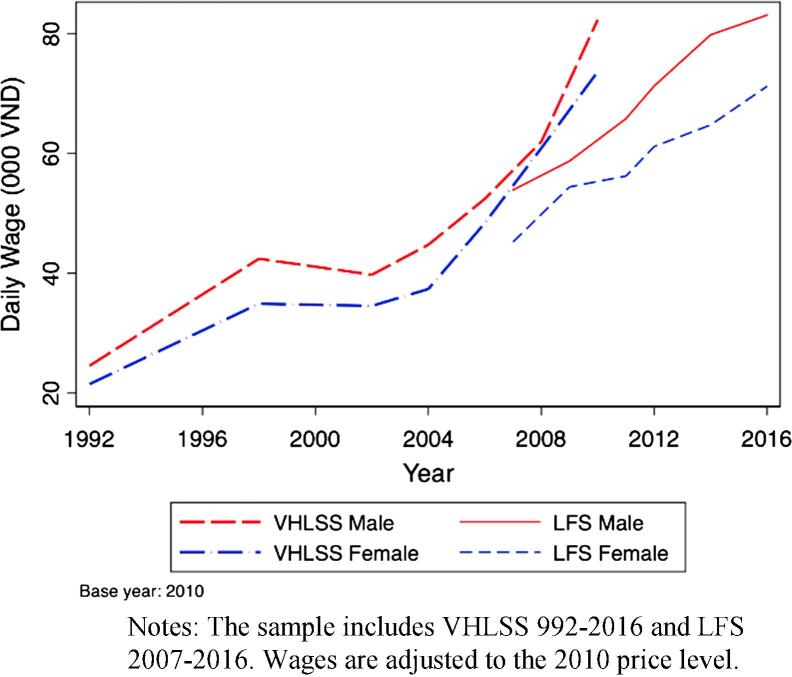
Median daily real male and female agricultural wage, 1992–2016. Notes: The sample includes VHLSS 992–2016 and LFS 2007–2016. Wages are adjusted to the 2010 price level.

Fig. 2 provides median daily real agricultural wages from the VHLSS and LFS data reported separately for women and men from 1992 to 2016. In the VHLSS, these daily wages more than quadrupled over this period, rising from 24,570 VND (or 1.32 USD) for men and 21,500 VND (or 1.15 USD) for women in 1992 up to 82,373 VND (or 4.42 USD) for men and 73,685 (or 3.96 USD) in 2010, according to the 2010 exchange rate of 18,612 VND per USD (World Bank 2019). This increase implies a very robust compound annualized growth rate of 7% for both men and women between 1992 and 2010. Fig. 2 also includes the complementary analysis for the LFS data from 2007 to 2016 which match trends in the VHLSS. These similar results across sampling procedures support the conclusion that there has been a robust increase in real agricultural wages over this period.^14^

Disparities between men and women remain relatively constant over both samples, with women suffering 11.4% and 14.2% wage penalties on average in the VHLSS and LFS, respectively. These disparities do not appear to vary systematically over time, reaching a minimum of 1.7% in 2008 in the VHLSS and a maximum of 18.9% in 2014 in the LFS. This gender pay gap is a bit lower than the global average of 15.6% (ILO, 2018). Is this pay gap due to different types of activities that women are involved in? The VHLSS data do not provide information on participation of different activities by gender however they identify gender-specific agricultural wages separately by task, as captured in Appendix Table A7. Women consistently received lower wages for the same tasks as men, pointing to gender inequality in returns to agricultural labor. Besides gender differences, these results also show little evidence of systematic changes in the harvest season wage premiums and suggest that any changing task composition likely has little role in driving observed increases in real agricultural wages.

**Fig. 3:**
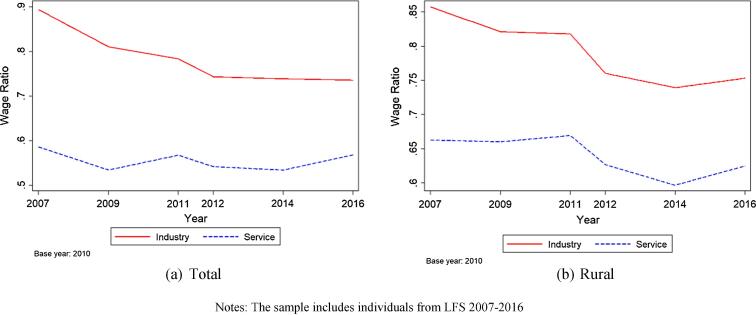
Ratio of agricultural to non-agricultural wages, 2007–2016. Notes: The sample includes individuals from LFS 2007–2016.

How do the rapid increases in wages relate to increases in wages in other sectors? Fig. 3 plots the ratio of agricultural wages to industrial wages and service sector wages over time and across both rural and urban areas in panel (a) and examining rural areas only in panel (b) for the LFS data. Despite the broad increases in real agricultural wages observed in Fig. 2, agricultural wages are increasingly exceeded by wages in the industrial sector while wages in service industries maintain a relatively stable, high ratio to agricultural wages. These findings are consistent with greater premiums to education in the non-farm sectors. Growing industrial wage disparities may help to explain increasing rates of urbanization and substitution away from agricultural towards nonagricultural employment. Comparing panels (a) and (b) of Fig. 3, we observe similar trends in both urban and rural areas. This is a pattern of intersectoral differentiation in the returns to labor, not a rural–urban difference. Continuous transition of the labor force from agriculture to nonfarm sectors, combined with increasing industrial sector-agriculture wage ratio, features a successful structural transformation in which “high-productivity employment opportunities (in nonfarm sectors) have expanded and structural change has contributed to overall growth” (McMillan and Rodrik 2011).

Table 4 examines the evolution of urban/rural real wage ratios overall as well as urban/rural real agricultural wages specifically. Panel A of Table 4 indicates a rapidly shrinking urban-to-rural wage ratio for both men and women, suggesting spatial convergence in labor markets. Panel B finds that agricultural wages evolved at similar rates in both urban and rural areas, with slight spatial convergence in female wage rates, but none for men. These results suggest agricultural labor market integration across regions, which is likely driven in part by the widespread seasonal rural–urban migration (de Brauw and Harigaya 2007). Migration from rural to urban areas during agricultural lean seasons tend to push up rural wages and thus lower urban/rural wage ratios.^15^

**Table 4:** Evolution in urban/rural real wage ratios over time.

		2007	2009	2011	2012	2014	2016
Panel A: Ratio of urban to rural real wage ratios over time							
	General	1.460	1.310	1.266	1.280	1.293	1.272
	Male	1.457	1.323	1.278	1.292	1.323	1.275
	Female	1.478	1.336	1.266	1.274	1.270	1.230
Panel B: Ratio of urban to rural real *agricultural* wage ratios over time							
	General	1.257	1.221	1.130	1.200	1.208	1.244
	Male	1.281	1.456	1.160	1.296	1.257	1.291
	Female	1.223	1.083	1.114	1.141	1.166	1.152

Notes: The sample includes individuals from LFS 2007–2016.

To examine whether particular regions may be driving trends in agricultural wages, Appendix Table A8 reports median real agricultural wages by gender as well as across the six main geographic regions. While there is substantial heterogeneity in wage levels across regions, all regions experienced similarly large increases in real agricultural wages over this period, with parallel changes across genders within each region. Consistent with findings in Table 4, wages appear to converge across regions over time with the coefficient of variation across regions dropping from 0.39 in 1992 to 0.13 to 2010 for men and 0.22 to 0.17 for women over the same interval.

One factor driving these agricultural wage increases over this period may be increasing minimum wages imposed by the national government under the general Labor Code. Minimum wages vary by region and sector and are typically adjusted annually. Unlike in some countries, minimum wage requirements apply to farms, households, cooperatives, in short to any individuals or organizations who employ workers. But enforcement is widely understood to be spotty and thus it is unclear how much compliance there is. One might naturally suspect that agricultural wages fall below the required minima or that the minimum wage rates set by government bind for farms and firms. But especially if minimum wages constrain agricultural employers, then minimum wages might help boost real wages, both by directly inducing higher wages for agricultural workers as well as indirectly, by increasing reservation wages throughout the economy.

Perhaps surprisingly, median and mean wage rates in agricultural consistently exceed the minimum wage rate.^16^ As shown in Panel A of Appendix Table A9, in 1992 average agricultural wages for both men and women were below the relevant minimum wage. But from 1998 to 2016 agricultural wages have consistently exceeded the minimum wage rates, by 17–119%, without any clear time trend. In Panel B, we see that the percentage of individual-specific wage rates that fell below the district-and-year-specific minimum wage has risen from 6.1% to 11.3% in the non-agriculture sectors and has risen significantly, from 14.5% to 28.4% nationwide, 2012–2016, in agriculture. So although mean and median agricultural wages steadily exceed minimum wages, it does appear that there has been increasing dispersion in agricultural wages, with the lowest wages not keeping pace with increases in region-specific minimum wages, especially among women (38.9% versus 22.9% among men in 2016) and in the more agricultural regions (those other than Red River Delta and South East). Minimum wage laws do not seem to be driving growth in real agricultural wages since noncompliance rates have been increasing in agriculture nationwide.

## 4 Evolution of the agricultural sector

Vietnam experienced a rapid transition over the 1992–2016 period, with large-scale movement of workers into non-farm employment, in rural as well as urban areas, and at sharply increasing real wage rates. What sort of transitions happened in the agricultural sector during this time, in particular do we see evidence of family farm consolidation due to labor exits, labor-saving factor substitution due to rising real agricultural wages in response to intersectoral labor market integration, and any erosion of small farms’ competitiveness within Vietnamese agriculture?

### 4.1 Family farm size distribution

Does family farmland become inevitably consolidate during a rapid rural transformation? The answer in the Vietnamese case is clearly no. Vietnamese agriculture rests on very small farm units; that has remained unchanged throughout the structural transformation. As shown in Appendix Figure A3, a median farm household cultivated 0.35 ha and 0.29 ha in 1992 and 2016, respectively, while the mean cultivated area was essentially unchanged, increasing very slightly from 0.57 ha to 0.61 ha from 1992 to 2016. The cultivated area of annual crops dropped from 0.31 ha in 1992 to 0.20 ha in 2016 for the median farm household while the mean cultivated area of the annual crops only slightly dropped from 0.49 ha to 0.43 ha during the same period. Therefore, there has been some modest substitution in land use, much of it towards use for livestock, reflecting rapid expansion in demand for animal source foods arising due to increasing incomes.

**Fig. 4:**
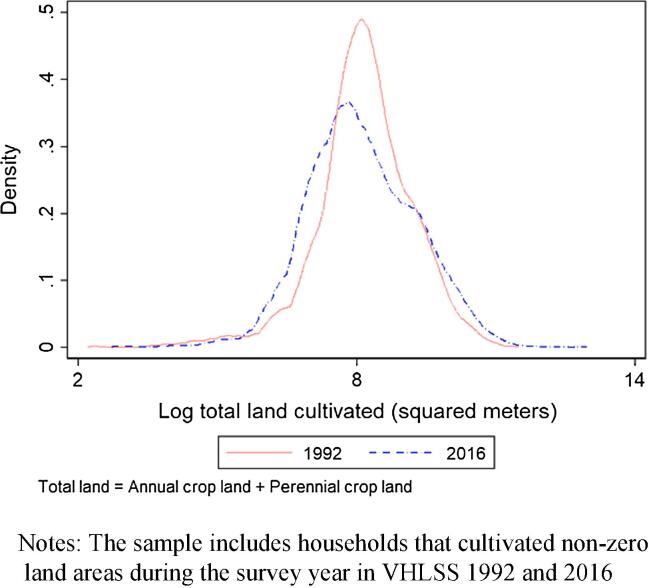
Distribution of total cultivated land per household in 1992 and 2016. Notes: The sample includes households that cultivated non-zero land areas during the survey year in VHLSS 1992 and 2016.

Fig. 4 offers a different view of the land distribution evolution, plotting the distribution of total cultivated land area per household in 1992 and 2016. Landholding has a single modal distribution in both rounds and is quite concentrated. This landholding structure likely results from egalitarian allocation during the period of central planned economies. Compared to 1992, the landholding distribution in 2016 is slightly less dispersed and has a slightly lower mode. We do not see any evidence of family farm consolidation. Farm sizes were small and remained small during Vietnam’s rapid structural transformation, despite the fact that the Government of Vietnam started its land reform in the late 1980 s, focusing on decollectivization that allocated secure land use rights to farm households under the 1993 Land Law (Ravallion and van de Walle 2008). Farmland consolidation may nevertheless have been deterred by the slow distribution of land use certificates in practice, limited duration of usufruct rights (20 years for annual crop plots according to the 1993 Law), and certain government regulations including the limits of annual cropland holding size and land use restrictions (Deininger and Jin, 2008, Do and Iyer, 2008, World Bank, 2016). Our finding is consistent with Tarp (2017) who also finds no evidence of inter-farm consolidation though there is some evidence in the VARHS data he uses of intra-farm consolidation (i.e., reduced number of plots within farm).^17^ .

Small farms have not ignited a significant increase in land rental markets either. As shown in Appendix Figure A4, the proportion of farm households that rent land in or out has remained low and relatively stable, at 10% or less throughout the period. We do see some modest convergence between reports of renting in and renting out land, which might reflect survey respondents’ increased willingness to report renting out land over time as the market-orientation of the economy became more firmly established, or could reflect the sectoral outmigration of workers from farm families, leading to small-scale rentals.

### 4.2 Mechanization

With small farm sizes, one might naturally expect that mechanization rates would have remained very low, given economies of scale in mechanization, especially for motorized equipment such as tractors, harvesters, combines, etc. (Binswanger, 1986, Foster and Rosenzweig, 2011, Foster and Rosenzweig, 2017; Otsuka et al., 2013; Yamauchi, 2016, Takeshima, 2017). Yet rapidly increasing real wages and the steady migration of workers out of agriculture call for the replacement of labor by machine. Which effect has dominated in determining the pattern of mechanization of Vietnamese agriculture: the farm size scale or the real wage substitution effect?

**Fig. 5:**
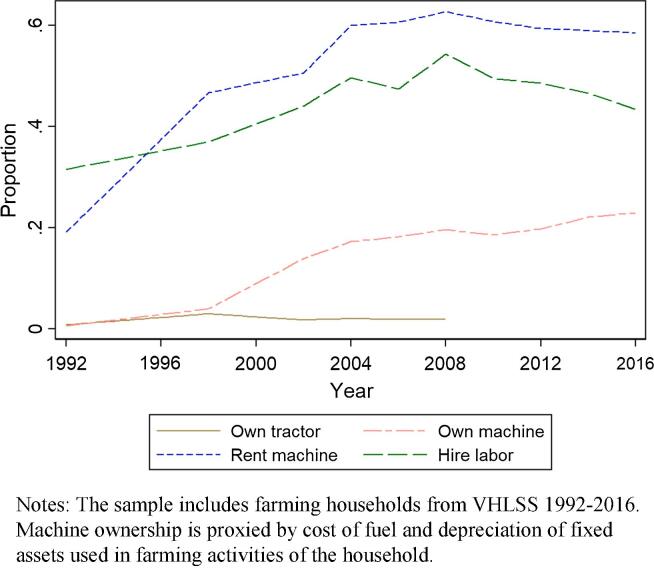
Proportion of farm households that owned tractors, owned any machinery, rented machinery, or hired labor. Notes: The sample includes farming households from VHLSS 1992–2016. Machine ownership is proxied by cost of fuel and depreciation of fixed assets used in farming activities of the household.

The answer, as in China, is that robust machine rental markets have emerged rapidly to address the scale-based, indivisibility problems in machinery and permit mechanization in the face of labor out-migration and rising real wages, enabling small farmers to benefit from machinery adoption (Zhang et al. 2017). Fig. 5 plots the proportion of households that owned tractors, owned any machinery, rented machinery, or hired labor from 1992 to 2016.^18^ We do not see much change in tractor ownership from 1992 to 2008 (such information is not available in the later rounds); the rate remains very low, which is not surprising given the small landholdings that dominate agriculture in Vietnam. Ownership of any machinery increased from 1 percent in 1992 to 23 percent in 2016. The percentage of cultivating households that rented in machines more than tripled, however, from 19 percent in 1992 to 59 percent in 2016. This observation mirrors recent findings from China (Yang et al. 2013). The percentage of households that hired labor also increased sharply, from 32 percent in 1992 to 43 percent in 2016.

**Fig. 6:**
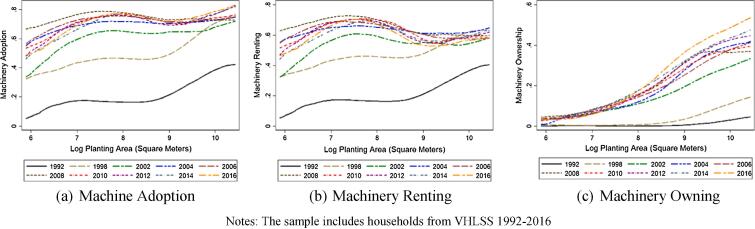
Nonparametric (local polynomial) regressions of machinery adoption, renting and owning conditional on farm size, over time. Notes: The sample includes households from VHLSS 1992–2016.

Fig. 6 plots the proportion of households adopting/using, renting and owning machinery conditional on farm size, from 1992 to 2016. Here we define machinery adoption as a binary variable which equals one if a household owns machinery or has incurred machinery rental expenses during the past year and zero otherwise. As expected, machinery ownership has been increasing in farm size throughout the sample period, reflecting economy of scale considerations. Machinery ownership has been steadily increasing over time throughout the entire farm size distribution, but increasing most and fastest among the largest farms, again consistent with economy of scale incentives.

The bigger story, however, is in machinery rental markets. In the early VHLSS rounds, 1992–2002, larger farmers had a far higher propensity to rent machinery. However, the machinery rental curves flatten in the later rounds, suggesting that smaller farmers have become almost equally likely to rent machinery in this decade. As a result, small farmers have machinery adoption rates almost as large as those of the biggest farm in later rounds. Clearly, rising real wage rates and/or reduced agricultural labor supply have compelled mechanization, even on the smallest farms where machinery ownership does not pay. Rental markets have emerged to fill the gap.^19^

### 4.3 Agrochemicals use

Agricultural modernization commonly involves increased use not only of machinery, but also of agrochemicals, both chemical fertilizers and pesticides (which include fungicides, herbicides, and insecticides). Fertilizer boosts crop and weed growth, stimulating demand for labor, while pesticides typically reduce labor demand by substituting for labor-intensive methods of pest eradication. Unlike machinery, there are no economies of scale to fertilizer or pesticide use. So the dominant drivers of agrochemicals uptake will typically be the profitability of use, which is driven both by crop and input prices and by real wage rates.

**Fig. 7:**
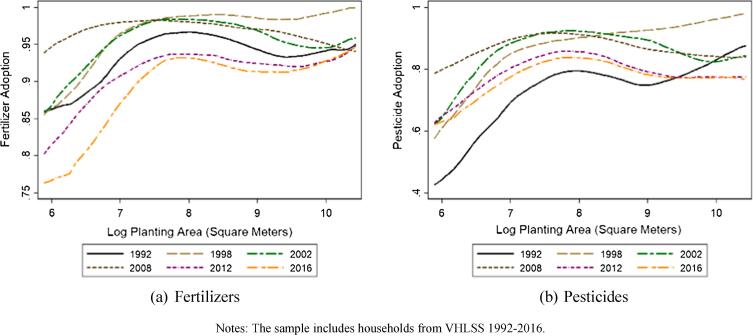
Nonparametric (local polynomial) regressions of agrochemical adoption conditional on farm size, over time. Notes: The sample includes households from VHLSS 1992–2016.

As reflected in Fig. 7, we see different patterns of use between fertilizers and pesticides. Labor-saving pesticide use has followed a pattern similar to that of machinery. In the earliest years of the VHLSS surveys, agrochemicals use was sharply increasing in farm size. The largest farms were more than twice as likely to use pesticides as the smallest farms. That relationship attenuated dramatically over time, so that by 2012 there was effectively no difference, with roughly 80% of farms of any size applying pesticides.^20^

By contrast, fertilizer use has been widespread through the 1992–2016 period. Back in the 1990s, 85% or more of farms of all sizes used chemical fertilizer. By 2012 and 2016, however, farms in the lower half of the farm size distribution were becoming less likely to apply fertilizer, leading to the emergence of a more pronounced, positive farm size-fertilizer use gradient. The data do not permit us to establish what caused the divergent paths in the fertilizer and pesticide gradients across the farm size distribution. But these patterns are consistent with farmer response to increasing real wage rates.

### 4.4 Land productivity

Along with labor, the main input in agriculture is land. And in Vietnam, the main crop is rice. Therefore, understanding the evolution of rice yields provides a useful indicator of the evolution of agricultural land productivity more broadly. It is possible that higher real wages lead farmers to apply less labor, leading to lower yields unless they compensate by using other inputs. We have just seen that Vietnamese farmers’ use of machinery and agrochemicals increased significantly over the 1992–2016 period. Did this offset any adverse yield effects arising due to the higher costs of agricultural workers?

**Fig. 8:**
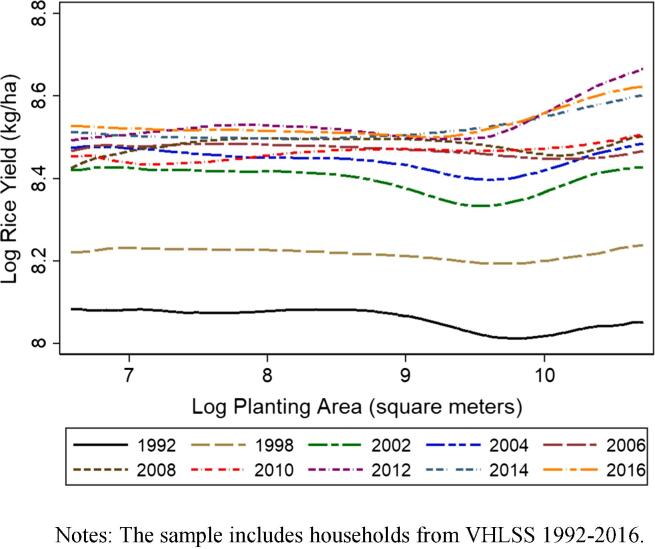
Nonparametric (local polynomial) regression of rice yield versus planting area, 1992–2016. Notes: The sample includes households from VHLSS 1992–2016.

Fig. 8 plots the locally weighted polynomial regression result of log rice yield against log planting area for each VHLSS survey round from 1992 to 2016. The sharp upward displacement of the yield curves between each successive survey round from 1992 to 2004 demonstrates the rapid growth in rice land productivity on farms of all sizes. Rice yields kept growing, though at a much slower and less stable pace, after 2004. The increased intersectoral integration of rural labor markets and the rise of real agricultural wages do not appear to have held back growth in rice yields.

It has long been observed in developing country agriculture that smaller farms are more productive per unit area cultivated than larger ones, on average (Chayanov, 1926/1986, Sen, 1962, Berry and Cline, 1979, Carter, 1984, Barrett, December 1996, Benjamin and Brandt, 2002, Barrett et al., 2010, Carletto et al., 2013). The dominant narrative behind the inverse relationship has historically been that multiple market failures can generate a size-productivity gradient even if the underlying technology of agricultural technology exhibits constant returns to scale (Feder, 1985, Barrett, December 1996). The evidence of such an inverse farm size–productivity relationship has often justified land policies favoring small landholders and deterring farm size expansion, as well as agricultural credit policies to promote smallholder access to commercial inputs.^21^

However, as a low-income agrarian economy undergoes rapid structural transformation, do factor markets for agricultural labor and machinery become more active, driving up real wages and attenuating the inverse relationship? Otsuka (2013) and Foster and Rosenzweig (2017) suggest that increasing real wages may reduce demand for agricultural labor, promote the use of machinery as a substitute for labor, and decrease any disadvantage larger farms might have once had, or even flip the inverse relationship to a direct relationship due to scale economies in machine use and access to financing. The 1992–2016 VHLSS panels offer an uncommon opportunity to test this intertemporal hypothesis. To our knowledge, a similar test has only been done in India based on a three-round household panel data (Deininger et al. 2018).

As described in section 4.1, we do not observe an increase in family farm size or consolidation of farms in Vietnam during dramatic structural transformation. Did small farms ever have a yield advantage? If so, could it be reversed without land consolidation? If so, this would further reinforce the story of increasingly integrated labor markets – as well as credit and machinery rental markets – that attenuate the inverse relationship.

To answer this question, we investigate the evolution of the inverse farm size-productivity relation using VHLSS panels from 1992/98 to 2014/16. We first estimate a rice yield equation using the five panels separately:

(1) $${\ln y}_{\mathrm{it}}=\alpha_{i}+\beta_{1}\ln h_{\mathrm{it}}+{\beta_{2}z}_{\mathrm{it}}+{\beta_{3}D}_{t}+\varepsilon_{\mathrm{it}},$$

where ${\ln y}_{\mathrm{it}}$ is log rice yield (in kilogram per hectare) for farm/household *i*, and year *t*; $\alpha_{i}$ is a household fixed effect which captures time invariant household and location-specific effects such as land quality and weather; $\ln h_{\mathrm{it}}$ is log rice planting area (in hectare); $z_{\mathrm{it}}$ is a vector of household-specific time-varying characteristics; $D_{t}$ is a year dummy which captures period-specific fixed effects (including interest rates, prices, and wages) that are common across communes; and $\varepsilon_{\mathrm{it}}$ is a random error term. The coefficient of interest, $\beta_{1},$ reflects the elasticity of rice yield with respect to planting area. A negative and statistically significant $\beta_{1}$ estimate supports the presence of an inverse relationship. If such a relationship lessened over time, the absolute value of $\beta_{1}$ will be smaller in a later panel than in an earlier panel. If such relationship is reversed, the $\beta_{1}$estimate will be positive.

Table 5 reports the regression results of Eq. (1) for the five panels. The dependent variable is rice yield aggregated over all rice varieties. We have two main findings. First, the coefficient estimate on planting area is statistically significantly negative in all panels, suggesting the existence of an inverse farm size–productivity relationship throughout the study period. This result is consistent with most of the published literature. Second, the estimated coefficient of planting area gets smaller (in absolute term) in later panels than the earlier panels. With a value of –0.036, the estimated coefficient of planting area in the most recent 2014/2016 panel is lower than that in the 1992/1998 (–0.155), the 2002/04 (–0.148), the 2006/08 (-0.066), and the 2010/12 panel (-0.079). Tests of the differences between the coefficient estimates in the 2014/16 panel and the previous panels show that the 2016/18 panel has a statistically significantly lower estimate of the size-productivity parameter than three out of the four previous panel rounds. The far lower explanatory power of the 2014/16 regression is also consistent with the idea that farm size and the other explanatory variables matter less over time.

**Table 5:** Regression Results on Land Productivity of Rice (All Varieties Included).

	1992/98	2002/04	2006/08	2010/12	2014/16	Difference	Difference	Difference	Difference
	(1)	(2)	(3)	(4)	(5)	(6)=(5)-(4)	(7)=(5)-(3)	(8)=(5)-(2)	(9)=(5)-(1)
Log total area of rice (all varieties)	−0.1554***	−0.1477***	−0.0664***	−0.0794***	−0.0360**	0.0434*	0.0304	0.1116***	0.1193***
	(0.0228)	(0.0235)	(0.0167)	(0.0171)	(0.0158)	(0.0233)	(0.0229)	(0.0283)	(0.0276)
Male household head	−0.0041	0.0663**	−0.0039	−0.0313	0.0130	0.0443	0.0170	−0.0532	0.0171
	(0.0317)	(0.0330)	(0.0326)	(0.0299)	(0.0341)	(0.0453)	(0.0472)	(0.0475)	(0.0465)
Age of household head	0.0001	0.0007	0.0003	−0.0003	0.0002	0.0005	−0.0002	−0.0005	0.0000
	(0.0011)	(0.0012)	(0.0010)	(0.0022)	(0.0011)	(0.0025)	(0.0015)	(0.0016)	(0.0016)
Highest education of household members	0.0127***	−0.0000	−0.0021	0.0045	0.0038	−0.0007	0.0059	0.0038	−0.0089*
	(0.0044)	(0.0046)	(0.0040)	(0.0044)	(0.0031)	(0.0054)	(0.0050)	(0.0055)	(0.0053)
Number of male members	0.0193	−0.0039	0.0082	−0.0045	0.0154	0.0199	0.0071	0.0193	−0.0039
	(0.0159)	(0.0139)	(0.0139)	(0.0155)	(0.0114)	(0.0192)	(0.0179)	(0.0180)	(0.0195)
Household size	0.0056	0.0156*	0.0035	0.0141*	−0.0068	−0.0208*	−0.0103	−0.0224*	−0.0123
	(0.0079)	(0.0095)	(0.0079)	(0.0083)	(0.0070)	(0.0109)	(0.0106)	(0.0118)	(0.0105)
Second year of the panel	0.0334***	0.0209***	0.0108***	0.0103	0.0018	−0.0085	−0.0090**	−0.0190***	−0.0315***
	(0.0028)	(0.0039)	(0.0030)	(0.0064)	(0.0029)	(0.0070)	(0.0042)	(0.0048)	(0.0040)
Observations	5917	4625	4333	3993	3782				
R-squared	0.205	0.080	0.029	0.098	0.010				

Notes: The sample includes households from VHLSS households in the 1992/98 panel, 2002/04 panel, 2006/08 panel, 2010/12 panel, and 2014/16 panel. Standard errors in parentheses, clustered at the commune level. The variable “Log total area of rice” centered around their sample means. * p < 0.10, ** p < 0.05, *** p < 0.01.

One reason these results may be biased is if the choice of rice varieties is correlated with farm size and if the productivity differs across rice varieties. We thus run the same regressions for spring ordinary rice and autumn ordinary rice separately as a robustness check. The 2002 VHLSS does not distinguish between these rice varieties; therefore, the 2002/2004 panel is left out of these analyses. The results, reported in Appendix Tables A10 and A11, are similar to those reported in Table 5, showing a significantly decreasing inverse size-productivity relationship for both spring and autumn rice over 1992/1998 and 2014/2016.

This change is associated with rising real wages and increasingly active machine rental and agricultural labor markets in rural Vietnam. As a result, the long-standing, labor-based productivity advantage assumed to exist among smaller farmers appears to have diminished altogether by the latter part of the period. Indeed, as real wages keep increasing, the inverse relationship may be reversed, leading to increased land concentration among farmers increasingly likely to employ machinery, without adverse effects on aggregate food production or prices.

### 4.5 Diversification of agricultural production

Just as many observers expect structural transformation to lead to farm consolidation, so too might one naturally expect rising incomes and enhanced market access have naturally led to diversification of agricultural production over time. We can explore this hypothesis by constructing a Herfindahl–Hirschman Index (HHI) for each farm household as

$${\mathrm{HHI}}_{i}=\sum_{j=1}^{8}S_{\mathrm{ij}}^{2}$$

where *i* indexes the farm household and *j* indexes each of eight categories of agricultural outputs: ordinary rice, glutinous rice, high-quality rice, other food crops, industrial crops, fruits, aquaculture, and livestock$.S_{\mathrm{ij}}^{\;}$ is the value share of output *j* of the total output value for farm *i*. HHI ranges from 0 to 1, with a higher value indicating lower diversification.

The top panel of Table 6 summarizes the HHI of agricultural output over the 2002–2016 period. Remarkably, the sector overall has exhibited decreased production diversity relative to the early 2000s. As seen in the bottom two rows, this effect is especially pronounced among the smallest farms. The largest quintile of farms, by land size, have seen some diversification. Appendix Table A12 summarizes the shares of agricultural output (by value) of the eight categories in 2002, 2010, and 2016. These results also point to a bifurcation in diversification patterns: the largest farms gradually diversified their output into other food crops and industrial crops; but the smaller farmers increasingly concentrated on rice production. We observe a high concentration of rice production in the 2010 survey round (which measured the 2009 harvest value) especially among smaller farms, in line with the sharp rise of rice prices during the 2008 global food price crisis. Neither larger nor smaller farms seem to diversify into livestock or horticulture significantly.

**Table 6:** Herfindahl–Hirschman Index (HHI) of agricultural output (by value).

		2002	2004	2006	2008	2010	2012	2014	2016
Total farms	Mean	0.426	0.417	0.475	0.501	0.498	0.466	0.461	0.468
	S.D	0.262	0.254	0.275	0.292	0.307	0.324	0.322	0.322
	Median	0.389	0.382	0.455	0.472	0.476	0.435	0.439	0.448
Smallest 20%	Mean	0.512	0.487	0.555	0.583	0.601	0.604	0.577	0.600
	S.D	0.256	0.261	0.274	0.281	0.294	0.318	0.319	0.309
	Median	0.479	0.452	0.516	0.541	0.577	0.582	0.558	0.569
Largest 20%	Mean	0.391	0.382	0.391	0.405	0.386	0.333	0.363	0.336
	S.D	0.329	0.312	0.337	0.356	0.356	0.354	0.358	0.348
	Median	0.314	0.323	0.319	0.315	0.297	0.192	0.250	0.216

Notes: The sample includes households from VHLSS 2002–2016. Smallest (largest) 20% refers to farms with cultivated land (annual crop land, perennial crop land, and water surface) falls in the bottom (top) 20%.

We note that the observed patterns can be partly attributed to the crop choice restrictions imposed by the government that require farmers to grow rice in certain plots for food security reasons. For example, farmers were obliged to grow rice on 35% of the total crop land area in 2006 (Markussen et al., 2011). Seasonal migration might play a role in explaining the higher production diversity in the early 2000s than that in recent years. De Brauw (2010) shows that migrant households tended to move out of labor-intensive rice production to more land-intensive crops due to lack of family labor for farming in 1990s. However, labor constraints may be partially relaxed as factor markets became more efficient in recent years. Therefore, the migration-induced effects on production diversity may be lower over time.^22^

While Vietnamese farms have not been diversifying their product mix appreciably over time, there has been a dramatic rise in farm households’ reliance on markets. Appendix Table A13 reports the share of food consumption expenditure coming from own production, i.e., autoconsumption. The median share of autoconsumption of own food production dramatically decreased from 0.535 in 1992 to 0.197 in 2016. This reflects sharply increased dependence on markets to source food, even among increasingly productive farm households. Rising rural incomes lead to more diverse diets, but with more efficient food markets, smaller farms have opted to concentrate on specific crops. This may reflect market-driven specialization according to comparative advantage, or the need to specialize in order to benefit from labor-saving mechanization that exhibits economies of scale. In contrast, larger farms may resort to higher diversification as a hedge against greater price risk exposure (Bellemare et al. 2013).

## 5 The evolution of well-being among rural households

**Fig. 9:**
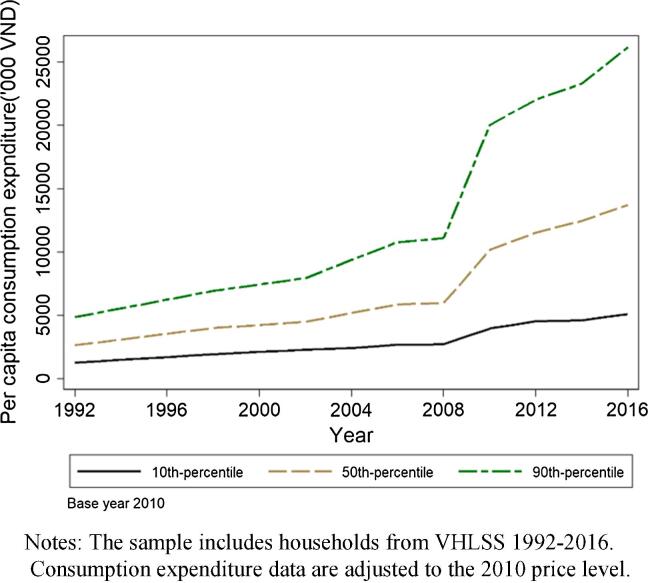
Evolution of rural real per capita expenditure per year (‘000 VND), 1992–2016. Notes: The sample includes households from VHLSS 1992–2016. Consumption expenditure data are adjusted to the 2010 price level.

As rural households have diversified out of agriculture, how has their well-being changed over time? Fig. 9 plots the 10, 50, and 90 percentiles of per capita real expenditure over the 1992–2016 period. The median per capita expenditure steadily increased from approximately 0.234 million VND (about USD 10.11) in 1992 to 1.221 million VND (about USD 52.75) per month in 2016 (based on the 2010 consumer price index). The 10 and 90 percentiles also increased steadily over time. But the 90 percentile grew more quickly than the median and the 10 percentile, suggesting increased wellbeing gap between the poorer and the richer rural households. Appendix Figure A5 plots the distribution of per capita consumption expenditure from 1992 to 2016 using VHLSS. Consistent with our observation from Fig. 9, The curve moved steadily and rapidly to the right over time, indicating improvement of well-being among rural households during the structural transformation period. The distribution also gets more dispersed in later rounds, pointing to increased variation in well-being among rural households.

To understand what factors are associated with household wellbeing and income sources over time, we regress the logarithm of household per capita consumption expenditures, share of income from agriculture, and share on income from wages on land and human capital endowments, the latter measured by years of schooling of the highest educated member of a household. We control for household demographics and regional fixed effects and cluster standard errors at the commune level. Since we do not control for several key relevant unobserved variables, our regression results should be interpreted as association rather than causality.

Table 7 reports that results for the first and last VHLSS rounds with available data to construct the key variables. The results from all feasible VHLSS rounds are reported in Appendix Tables 14–16. In Table 7 Columns (1)-(6) and Appendix Tables 14–15, we look at how landholding and human capital are associated with income shares from agriculture and from wages, respectively. Not surprisingly, landholding is positively associated with the household income share from agriculture and negatively associated with the share from labor earnings. Education is negatively associated with the income share from agriculture and positively associated with that from wages. The coefficient estimate relating education (years of schooling) to income share from wage increased steadily, from 0.0086 in 1992 to 0.0211 in 2016, suggesting that better educated rural households rely more on labor markets as an income source over time. The younger labor force is also better educated and more likely to migrate from agriculture to nonfarm sectors (largely through seasonal migration from rural to urban), leaving a more severely aging agricultural labor force than the nonfarm sectors. Not surprisingly, as we observed earlier, rural households now earn more from labor markets than from agriculture.

**Table 7:** Regression results of per capita consumption expenditure, and income share from agriculture and from wage.

	**Share of agricultural income**			**Share of wage income**			**Log per capita expenditure**		
	2004	2016	2016–2004	2004	2016	2016–2014	1992	2016	2016–1992
	(1)	(2)	(3)	(4)	(5)	(6)	(7)	(8)	(9)
Log of total land owned (square meters)	0.1299***	0.1311***	0.0012	−0.0836***	−0.0981***	−0.0145*	0.0638***	0.0154	−0.0485**
	(0.0053)	(0.0052)	(0.0074)	(0.0051)	(0.0056)	(0.0076)	(0.0171)	(0.0098)	(0.0197)
Highest grade completed of household members	−0.0180***	−0.0104***	0.0076***	−0.0635***	−0.0605***	0.0029	0.0513***	0.0736***	0.0223***
	(0.0015)	(0.0018)	(0.0023)	(0.0112)	(0.0145)	(0.0184)	(0.0058)	(0.0037)	(0.0069)
Male household head	0.0568***	0.0625***	0.0057	−0.0017***	−0.0026***	−0.0009*	0.0010	0.0446*	0.0436
	(0.0103)	(0.0119)	(0.0157)	(0.0003)	(0.0004)	(0.0005)	(0.0225)	(0.0237)	(0.0326)
Age of household head	−0.0017***	−0.0007**	0.0009**	0.0086***	0.0211***	0.0124***	0.0067***	0.0039***	−0.0028***
	(0.0003)	(0.0003)	(0.0004)	(0.0015)	(0.0019)	(0.0024)	(0.0007)	(0.0006)	(0.0009)
Number of male members	−0.0075	−0.0131**	−0.0057	0.0260***	0.0292***	0.0032	0.0120	0.0009	−0.0111
	(0.0049)	(0.0062)	(0.0079)	(0.0050)	(0.0076)	(0.0091)	(0.0077)	(0.0111)	(0.0135)
household size	−0.0056	−0.0140***	−0.0084	0.0154***	0.0271***	0.0117*	−0.0802***	−0.1651***	−0.0849***
	(0.0035)	(0.0041)	(0.0053)	(0.0034)	(0.0051)	(0.0061)	(0.0061)	(0.0081)	(0.0101)
Observations	4899	4626		4899	4626		3513	4626	
R-squared	0.236	0.313		0.112	0.173		0.266	0.366	

Notes: The sample includes households from VHLSS 1992–2016. Standard errors in parentheses, clustered at the commune level. Regional dummies are included in all regressions. * p < 0.10, ** p < 0.05, *** p < 0.

Columns (7)-(9) of Table 7 and Appendix Table 16 present results on consumption expenditure. Both landholdings (owned land) and education are positively associated with consumption in all rounds. However, the coefficient of landholding becomes smaller over time, even becoming statistically insignificantly different from zero in the 2016 round, while the coefficient of education becomes larger and more significant over time. These results reinforce the earlier findings that although rural households in Vietnam have remained engaged in farming, they are increasingly dependent on the returns to human capital in labor markets and depend less today than previously on landholdings to support their well-being. Although agricultural productivity has increased sharply over time, the large improvements observed in rural well-being (Fig. 9) appear most strongly associated with improvements in human capital remunerated in labor markets increasingly integrated across sectors and space. Indeed, as shown in Appendix Figure A6, rural household expenditure is positively correlated with income share from wages and negatively correlated with income share from agriculture for all rounds. Our finding also suggests that, for rural households, human capital accumulation (rather than land endowment) is an essential means of successful transformation.

## 6 Conclusions

Vietnam’s dramatic structural transformation over the past generation offers an uncommon glimpse into the path followed as a low-income agrarian economy grows rapidly. In 1992, Vietnam looked remarkably comparable to current day Liberia in terms of per capita income, share of output and employment in agriculture, reliance on rice and cassava as staple crops, etc. Today it continues to grow at a rapid rate (6–7% annually), diversifying and creating jobs quickly, and transforming into an increasingly urban and non-farm lower middle-income economy. Several key patterns of Vietnam’s structural transformation merit comment as they relate to prospective futures for today’s low-income agrarian economies.

First, the direct employment creation potential of agriculture, especially for youth, is limited. The agricultural labor force is slowly shrinking and aging more rapidly than is the labor force as a whole. Even farming families are diversifying out of agriculture, increasingly earning more of their total household income from the non-farm sector. Youth are increasingly well educated, enjoying a wider array of remunerative non-farm job options than their parents did. Meanwhile, the endogenous changes in agriculture, especially mechanization and uptake of labor-savings inputs such as pesticides, relax farm households’ labor constraints, freeing young people to seize non-farm opportunities.

Second, real wage convergence between rural–urban regions has gone hand-in-hand with increased diversification of the rural economy into the non-farm sector nationwide and rapid advances in educational attainment in all sectors’ and regions’ workforce. This enhanced integration also manifests in steady attenuation of the longstanding inverse farm size-yield relationship, which only exists when there exist multiple rural market failures. Minimum wage restrictions do not seem to explain growth in real agricultural wages. Indeed, while compliance with minimum wage laws appears quite high in the non-farm sector, noncompliance in the agriculture sector has been increasing this decade, especially in the most agriculturally dependent regions. Minimum wage laws have not prevented a widening in the intersectoral wage differential, which likely reflects differing returns to human capital, particularly educational attainment.

Third, there is no indication of significant disinvestment of households from farmland nor of significant growth in agricultural labor demand nor the growth of a farmworker population. Indeed, the family farmland distribution has remained largely unchanged over these 24 years, as has the share of workforce earning wages in agriculture. There has been no farm consolidation and no appreciable diversification out of rice production. Although this precludes seizing economies of scale, thanks to the emergence of robust machinery rental markets it has not obstructed mechanization, nor the uptake of labor-saving pesticides. Rice yields increased rapidly in the earlier years, more slowly over the past decade. But farm households have clearly become better integrated into commercial marketing channels, as reflected in the sharp decrease in the share of food autoconsumed from home production.

Fourth, nonfarm sectors have been providing high-productivity employment opportunities, which is a driving force contributing to wellbeing improvement among rural households. As rural households rely more heavily on the labor market, human capital accumulation (rather than land endowment) is an essential means for rural households to benefit from successful transformation.

Will today’s low-income agrarian economies necessarily follow the path Vietnam has taken over this past quarter century? That seems unlikely, given the many context-specific features that have guided Vietnamese development over the past generation. Nonetheless, there are important lessons to be learned from the experience of one of the world’s most rapidly transforming rural economies.

## CRediT authorship contribution statement

**Yanyan Liu:** Conceptualization, Methodology, Formal analysis, Writing - original draft. **Christopher B. Barrett:** Conceptualization, Methodology, Writing - review & editing. **Trinh Pham:** Formal analysis, Data curation. **William Violette:** Formal analysis, Writing - review & editing.

## Footnotes

1 See Timmer (1993) for the policy context for the government's reforms to agriculture and the rural economy as the starting point of our analysis.

2 See also the papers in recent special journal issues on the topic of structural transformation, such as *Agricultural Economics* (48, S1; 2017), *World Development* (105; 2018), or *Journal of Development Studies* (54, 5; 2018).

3 For example, McCaig and Pavcnik (2018) demonstrate that the US-Vietnam Bilateral Trade Agreement boosted labor productivity in manufacturing, inducing a reallocation of workers into formal manufacturing.

4 Throughout this paper, agricultural income is referred to agricultural revenue or gross income from agriculture with no deduction of input costs.

5 Our data do not provide information on commercial farms. The farm size in this paper refers to family farm size.

6 The shrinking rural–urban wage gap is also found in Benjamin et al. (2017) using VHLSS 2002–2014.

7 Although the GSOV claims the data are representative of the country, households in surveyed communes are on average better off than households in other communes (Hansen and Le, 2013). This has created some concern that the sample selection deviated from a fully random approach at some point(s), although these differences are not substantial.

8 As is apparent in Table 1, the 1992–98 VLSS sampled fewer communes and more households/commune than the subsequent 2002–2016 VHLSS.

9 See Appendix A for description of the sampling framework.

10 A household is considered as an agricultural/farming household or a household engaged in agriculture if any member had been worked in agriculture (including forestry and aquaculture) during past 12 months before each survey time.

11 Authors’ calculations based on VHLSS household data.

12 Please see Appendix B for more detailed discussion.

13 Note that the absence of a rural–urban difference may also reflect the census’ practice of counting many rural–urban migrants as rural population due to the Vietnam’s restrictive residential registration system (*ho khau*).

14 Results for mean wages included in Appendix Figure A2 exhibit almost identical patterns to median wage results in Fig. 2.

15 Seasonal migration is not a focus of this study due to data limitations, though it likely plays an important role in structural transformation in Vietnam.

16 There are four minimum wage “regions” in Vietnam into which each (sub-provincial) district is categorized. Before 2012, there were separate minimum wage schedules for domestic and foreign firms. In 2012, the two were merged into a single schedule. The minimum wage rates (‘000 VND) for four regions for 2012, 2014, and 2016 ranged from (1400, 1900,2400) to (2000, 2700, 3500), reflecting differential rates of minimum wage growth across space.

17 See Tarp (2017) for a more detailed description on land policy.

18 Because the VHLSS survey instruments do not consistently list all agricultural machinery, machine ownership is proxied by cost of fuel and depreciation of fixed assets used in farming activities of the household, as those are included consistently throughout the survey years.

19 Promoted by the strategy for investing in agricultural mechanization under the centrally planned economy, tractor adoption rates in Vietnam reached moderately high levels by 1980 (close to 30 percent of the entire country and even higher in southern Vietnam). Such exposures to mechanization likely have contributed to the resurgence of tractors in the 1990s. Most low-income countries have not been exposed to such high-level of machinery adoption as Vietnam and are likely faced with more demand constraints (Takeshima et al., 2021).

20 Pesticides can also pose human health risks, especially to agricultural workers. Research in southeast Asian rice systems has previously established increased human health costs associated with expanded uptake of pesticides (Antle and Pingali, 1994) and recent survey data from sub-Saharan Africa establish similar correlations as pesticide use increases in some parts of that region (Sheahan et al. 2017). Ecological and health costs of excessive pesticide use have been a growing concern in Vietnam (Dasgupta et al. 2007). We leave exploration of the pesticide-health relationship in Vietnamese agriculture to future work.

21 A recent literature suggest that the inverse farm size-productivity relationship appears attributable to measurement error in crop output in multiple data sets from Africa (Gourlay et al. 2017, Desiere and Jolliffe 2018, Abay et al. 2019). Since there is no reason to expect an intertemporal trend in such measurement error in the VLSS data and yet we see a clear trend in the size-productivity relationship parameter, we suggest that the attenuation we observe is at least partly real, not merely an artefact of measurement error.

22 We are not able to test this hypothesis due to data limitations.
