# Supplementary material for: The intertemporal evolution of agriculture and labor over a rapid structural transformation: Lessons from Vietnam
Source: Food Policy. 2020 Jul;94:101913. doi: 10.1016/j.foodpol.2020.101913 (PMC7398042; doi:10.1016/j.foodpol.2020.101913)
Supplement: Supplementary data 1 [file mmc1.docx]

**Online Appendices to “The Intertemporal Evolution of Agriculture and Labor over a Rapid Structural Transformation: Lessons from Vietnam”**

Yanyan Liu, Christopher B. Barrett, Trinh Pham, William Violette

April 2020 revision

**Appendix A: Labor Force Survey Sampling Framework**

The LFS sample is randomly selected in a two-stage stratification design. In the first stage, each centrally governed city or province constitutes a main stratum, which is divided into two sub-stratums representing Population and Housing Census enumeration areas in rural and urban areas. Enumeration areas are then randomly selected using the Kish method. In the second stage, 15-20 households were selected from each sub-stratum enumeration area, yielding a sample that is statistically representative at the national, urban/rural, and six regional levels. The LFS 2007 was sampled based on the 1999 Population and Housing Census. The LFS 2009-14 used the 2009 Census as their sampling frame. The LFS 2015-16 sample is based on the 2014 Intercensal Population and Housing Survey. Unlike other rounds which were conducted over 12 months of the respective year, the 2009 round was conducted in September only. For LFS 2011-16, the sample was selected under the 2-2-2 rotation mechanism, under which each enumeration area was divided into two rotation groups, households of each rotation group were surveyed in two consecutive quarters, excluded from the next two consecutive quarters, and again included in the following two consecutive quarters. Each enumeration area was selected in the sample at a maximum of four times a year.

**Appendix B: Evolution of Hired Agricultural Labor**

Vietnamese agriculture is traditionally dominated by farmers who cultivate their own land. This pattern remained largely unchanged during the structural transformation. As shown in Appendix Table A5, farmworker households (i.e., households that are employed in agriculture but do not own livestock or farmland) remained just 2-5% of all households from 2002 to 2016, with no discernible increase in the share of landless farmworker households. Rapid growth in agricultural productivity (see section 4) has not translated into increased employment in agriculture, nor to the rise of a landless farmworker population. This may stem from the lack of farm consolidation (see section 4).

In Appendix Table A6 we see that hired agricultural workers increasingly earn hourly or daily wages, growing from 57 to 71% of all payment methods from 2007-2016. There was a corresponding, modest fall in the rate of piece rate work, from 25 to 17%, and in in-kind payments, which declined from 4.5% in 2007 to essentially disappear in more recent years. The share of salaried workers has remained constant at just 11%. This underscores further how much Vietnamese agriculture continues to depend on family-based production units.

**Appendix Figures and Tables**

**Figure A1. Population pyramid of Vietnam 1989, 1999, 2009**

| 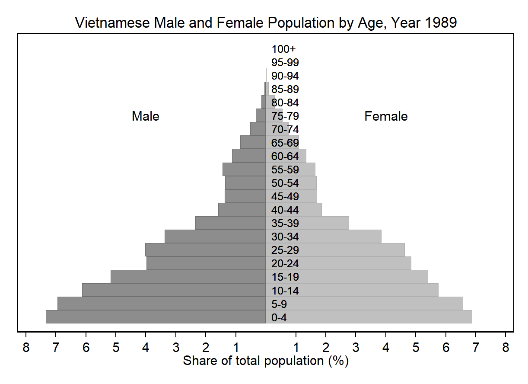 | 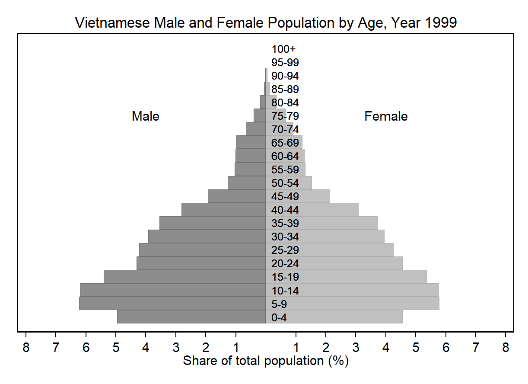 | 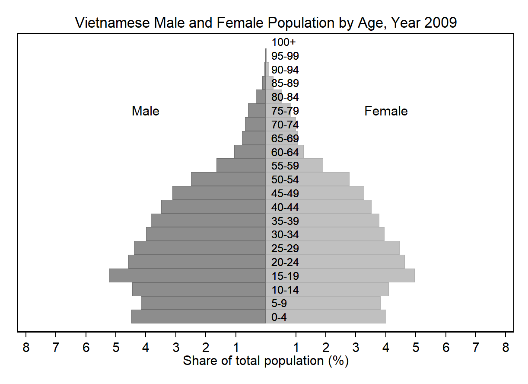 |
| --- | --- | --- |
| 1. Total population | | |
|  | | |
| 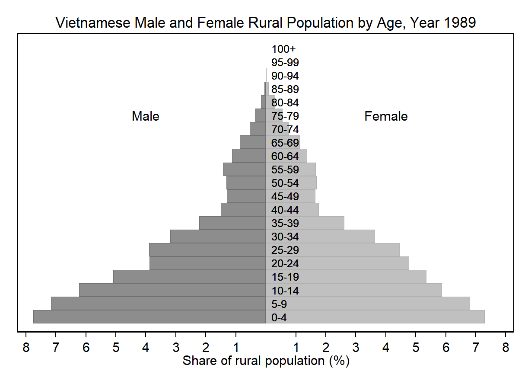 | 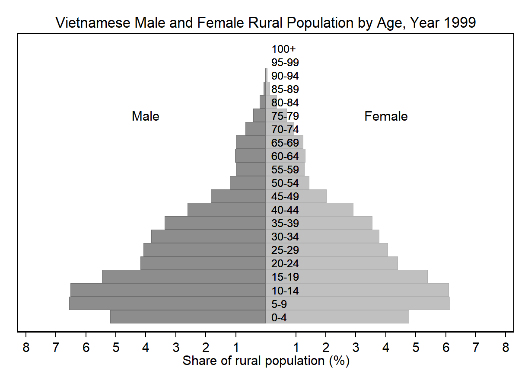 | 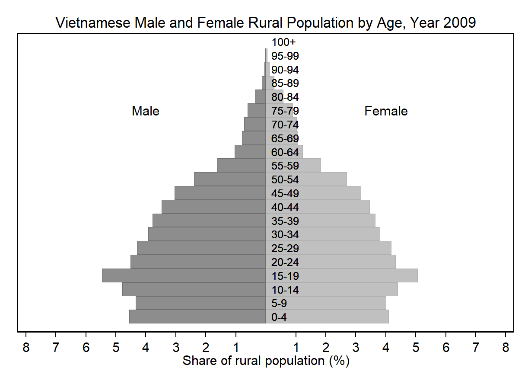 |
| 1. Rural population | | |

Notes: The sample includes 5%, 3%, and 15% of the Population and Housing Census 1989, 1999, and 2009, resepectively

**Figure A2. Mean daily real male and female agricultural wage, 1992-2016**


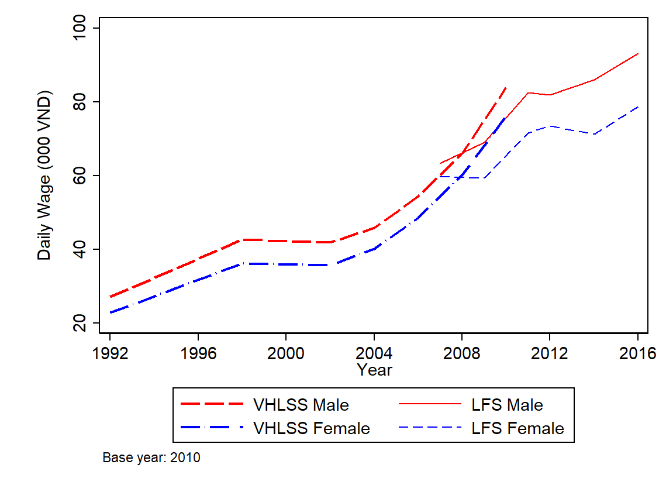


Notes: The sample includes households from VHLSS 1992-2016 and individuals from LFS 2007-2016.

**Figure A3. Trend of total land cultivated per household and total annual crop land cultivated per household, 1992-2016**


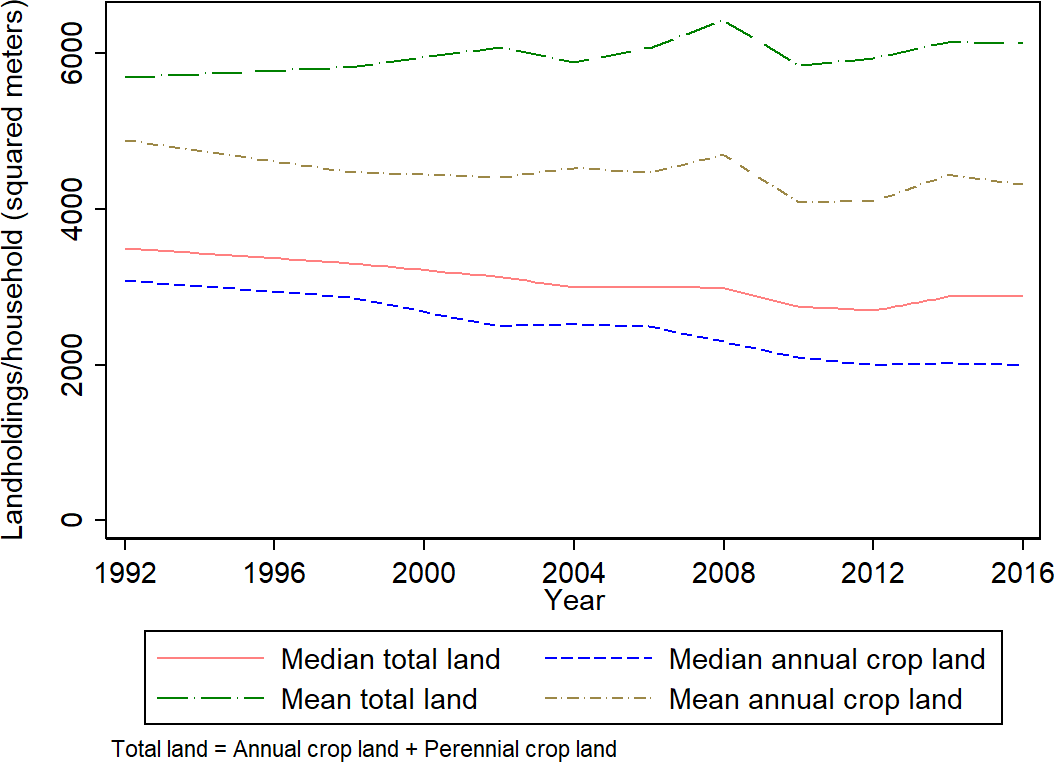


Notes: The sample includes households from VHLSS 1992-2016.

**Figure A4. Proportion of farm households that rent in/out land, 1992-2016**

Notes: The sample includes households from VHLSS 1992-2016.

**Figure A5. Distribution of per capita expenditure in 1992, 2002, 2012, and 2016**


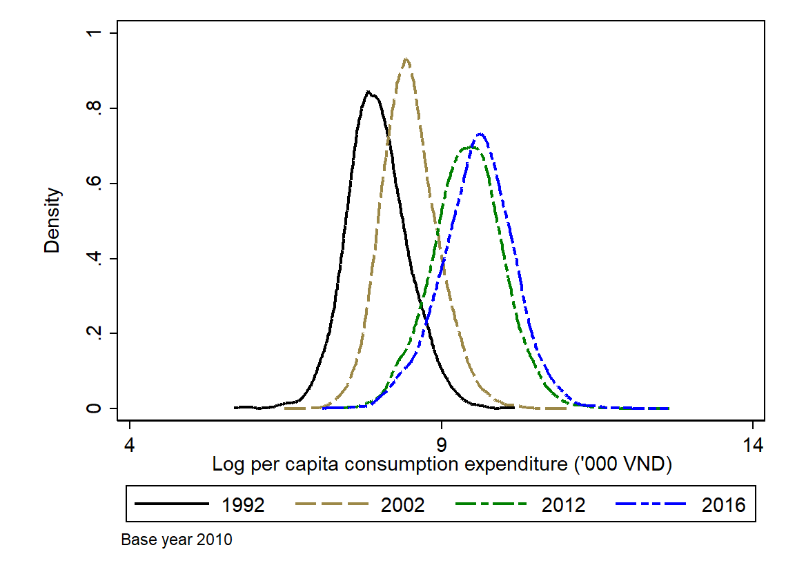


Notes: The sample includes households from VHLSS 1992, 2002, 2012, and 2016. The expenditure data are adjusted to the 2010 price level.**Figure A6.** **Income Share from Agriculture and from Wage of Rural Households**

| 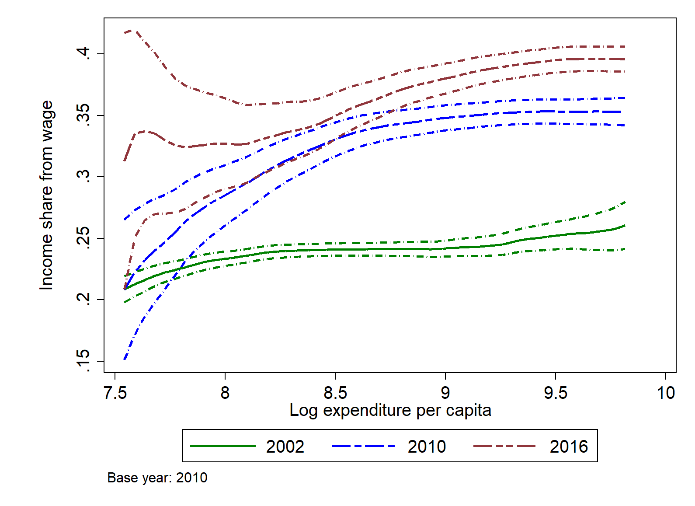 | 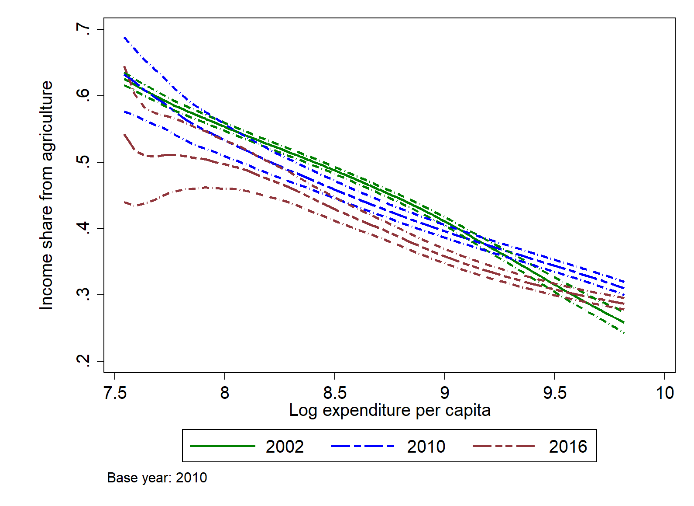 |
| --- | --- |

Note: Dashed lines denote 95% CI.

**Table A1. Description of VHLSS samples**

| Panel | Total | | Rural only | |
| --- | --- | --- | --- | --- |
|  | Number of households | Number of communes | Number of households | Number of communes |
| 1992-1998 | 4,303 | 147 | 3,397 | 115 |
| 2002-2004 | 4,081 | 1,409 | 3,148 | 1,084 |
| 2006-2008 | 4,085 | 1,502 | 3,080 | 1,118 |
| 2010-2012 | 4,157 | 1,524 | 3,064 | 1,108 |
| 2014-2016 | 4,266 | 1,544 | 3,035 | 1,082 |

**Table A2. Description of LFS samples**

| Survey round | Total individuals (persons) | Rural individuals (persons) | Estimated share of rural individuals (%) | Estimated labor force participation rate (%) | |
| --- | --- | --- | --- | --- | --- |
|  |  |  |  | Total | Rural |
| 2007 | 496,842 | 182,656 | 70.46 | 80.78 | 83.95 |
| 2009 | 49,431 | 21,729 | 69.24 | 82.42 | 85.03 |
| 2011 | 842,092 | 334,663 | 67.21 | 82.84 | 86.20 |
| 2012 | 562,168 | 288,426 | 66.71 | 82.98 | 85.98 |
| 2014 | 560,642 | 286,797 | 66.89 | 84.18 | 87.25 |
| 2016 | 615,986 | 348,445 | 64.72 | 83.52 | 86.96 |

Notes: Individuals aged 15 and above are included in the sample, otherwise stated. Labor force participation rate is the proportion of the population ages 15-60 that is economically active (employed, under-employed, un-employed). Estimation for share of rural individuals and labor force participation rate uses sampling weights available in the dataset.

**Table A3. Agricultural Labor Force and Households Involved in Agriculture**

|  | 2007 | 2009 | 2011 | 2012 | 2014 | 2016 |
| --- | --- | --- | --- | --- | --- | --- |
| Panel A: Share of agricultural labor force in total | | | |  |  |  |
|  | 0.484 | 0.463 | 0.475 | 0.466 | 0.457 | 0.394 |
| By region |  |  |  |  |  |  |
| Central Highlands | 0.672 | 0.707 | 0.727 | 0.703 | 0.718 | 0.691 |
| Mekong River Delta | 0.516 | 0.491 | 0.504 | 0.512 | 0.500 | 0.455 |
| North and South-Central Coast | 0.698 | 0.673 | 0.669 | 0.669 | 0.657 | 0.587 |
| North East and North West | 0.535 | 0.553 | 0.552 | 0.539 | 0.531 | 0.451 |
| Red River Delta | 0.404 | 0.339 | 0.368 | 0.347 | 0.338 | 0.272 |
| South East | 0.201 | 0.216 | 0.203 | 0.193 | 0.178 | 0.127 |
|  |  |  |  |  |  |  |
| Panel B: Share of agricultural labor force in rural areas | | | |  |  |  |
|  | 0.617 | 0.593 | 0.613 | 0.605 | 0.592 | 0.530 |
|  |  |  |  |  |  |  |
| Panel C: Agricultural labor force (Mil. Persons) in total | | | |  |  |  |
|  | 22.753 | 22.827 | 24.519 | 24.348 | 24.571 | 21.406 |
|  |  |  |  |  |  |  |
| Panel D: Agricultural labor force (Mil. Persons) in rural areas | | | |  |  |  |
|  | 21.361 | 20.940 | 22.266 | 22.026 | 22.293 | 19.598 |

Notes: The sample includes individuals from LFS 2007-2016.

**Table A4. Median Share of Agricultural and Wage Income in Total Household Income**

|  |  | 2002 | 2004 | 2006 | 2008 | 2010 | 2012 | 2014 | 2016 |
| --- | --- | --- | --- | --- | --- | --- | --- | --- | --- |
| Central Highlands | Agriculture | 0.731 | 0.665 | 0.664 | 0.625 | 0.587 | 0.627 | 0.583 | 0.521 |
|  | Wage | 0.076 | 0.056 | 0.106 | 0.094 | 0.215 | 0.138 | 0.175 | 0.220 |
| Mekong river delta | Agriculture | 0.412 | 0.373 | 0.331 | 0.212 | 0.248 | 0.208 | 0.231 | 0.191 |
|  | Wage | 0.163 | 0.104 | 0.130 | 0.134 | 0.192 | 0.232 | 0.231 | 0.251 |
| North East and North West | Agriculture | 0.689 | 0.615 | 0.580 | 0.603 | 0.520 | 0.491 | 0.477 | 0.410 |
|  | Wage | 0.000 | 0.071 | 0.080 | 0.071 | 0.127 | 0.207 | 0.224 | 0.251 |
| North and Central Coast | Agriculture | 0.475 | 0.431 | 0.389 | 0.377 | 0.305 | 0.251 | 0.234 | 0.228 |
|  | Wage | 0.000 | 0.063 | 0.104 | 0.094 | 0.248 | 0.279 | 0.322 | 0.331 |
| Red river delta | Agriculture | 0.411 | 0.341 | 0.299 | 0.278 | 0.187 | 0.147 | 0.119 | 0.078 |
|  | Wage | 0.117 | 0.156 | 0.174 | 0.229 | 0.384 | 0.379 | 0.431 | 0.445 |
| South East | Agriculture | 0.172 | 0.176 | 0.158 | 0.085 | 0.010 | 0.011 | 0.010 | 0.023 |
|  | Wage | 0.316 | 0.300 | 0.315 | 0.371 | 0.480 | 0.497 | 0.516 | 0.547 |

Notes: The sample includes households from VHLSS 2002-2016. The pattern is qualitatively identical in means.

**Table A5. Emergence of Farmworker Population**

|  | 1992 | 1998 | 2002 | 2004 | 2006 | 2008 | 2010 | 2012 | 2014 | 2016 |
| --- | --- | --- | --- | --- | --- | --- | --- | --- | --- | --- |
| Share of households employed in farming who do NOT own livestock nor cultivate land themselves, i.e., pure farmworkers | | | | | | | | | | |
|  |  |  | 0.027 | 0.015 | 0.019 | 0.050 | 0.027 | 0.031 | 0.028 | 0.028 |
|  |  |  |  |  |  |  |  |  |  |  |
| Number of households employed in farming who do NOT own livestock nor cultivate land themselves, i.e., pure farmworkers (thousand) | | | | | | | | | | |
|  |  |  | 355 | 201 | 266 | 722 | 375 | 451 | 407 | 444 |
|  | | | | | | | | | | |

Notes: The sample includes households from VHLSS 1992-2016. Estimates from LFS are similar, especially after 2012.

**Table A6. Payment Terms for Hired Agriculture Labor**

|  | Total | | | Rural | | |
| --- | --- | --- | --- | --- | --- | --- |
|  | 2007 | 2014 | 2016 | 2007 | 2014 | 2016 |
| Salaried | 11.1 | 11.18 | 11.34 | 9.13 | 10.17 | 10.06 |
| Per hour or per day | 57.28 | 68.18 | 70.95 | 58.47 | 69.15 | 71.45 |
| By piece | 25.4 | 19.61 | 16.56 | 26.04 | 19.6 | 17.43 |
| Others | 6.22 | 1.03 | 1.15 | 6.36 | 1.08 | 1.06 |
| In which: |  |  |  |  |  |  |
| In-kind | 4.47 | 0.05 | - | 4.52 | 0.06 | - |
| Unpaid | 0.98 | 0.43 | - | 1.03 | 0.42 | - |
| Others | 0.77 | 0.54 | - | 0.81 | 0.6 | - |

Notes: The sample includes individuals from LFS 2007, 2014, and 2016. -: data not available.

**Table A7** **Median daily real wage in 000 constant 2010 VND by gender and by task**

|  |  | 1992 | 1998 | 2002 | 2004 | 2006 | 2008 | 2010 | 2012 | 2014 | 2016 |
| --- | --- | --- | --- | --- | --- | --- | --- | --- | --- | --- | --- |
| Male | Land preparation | 29.011 | 43.743 | 43.353 | 48.564 | 56.472 | 68.064 | 84.512 | 97.905 | 108.779 | 117.984 |
|  | Crop planting | 23.209 | 41.946 | 39.497 | 44.021 | 51.341 | 61.785 | 79.939 | 94.018 | 104.639 | 104.125 |
|  | Crop tending | 21.499 | 38.427 | 38.439 | 38.851 | 48.404 | 61.073 | 73.948 | 83.028 | 94.275 | 102.562 |
|  | Crop harvesting | 24.570 | 43.743 | 41.469 | 48.564 | 57.968 | 70.546 | 84.512 | 99.633 | 109.199 | 119.317 |
| Female | Land preparation | 22.101 | 34.246 | 37.797 | 37.374 | 48.404 | 60.895 | 73.948 | 84.125 | 94.639 | 101.076 |
|  | Crop planting | 21.099 | 33.247 | 32.515 | 36.791 | 48.404 | 60.895 | 72.259 | 83.028 | 89.877 | 100.580 |
|  | Crop tending | 18.579 | 32.181 | 31.102 | 35.823 | 42.825 | 54.966 | 65.355 | 79.724 | 85.921 | 97.722 |
|  | Crop harvesting | 21.888 | 36.224 | 38.544 | 43.760 | 50.711 | 61.785 | 78.593 | 92.607 | 101.852 | 104.125 |

Source: Estimation from VHLSS 1992-2016

**Table A8. Median real agricultural wages across gender and six main geographic regions**

| **Male** | **1992** | **1998** | **2002** | **2004** | **2006** | **2008** | **2010** | **2012** | **2014** | **2016** |
| --- | --- | --- | --- | --- | --- | --- | --- | --- | --- | --- |
| Central Highlands | 25.68 | 36.83 | 37.80 | 39.62 | 49.69 | 70.55 | 74.94 | 92.61 | 101.85 | 99.43 |
| Mekong river delta | 43.52 | 53.56 | 49.37 | 53.73 | 60.51 | 67.96 | 84.51 | 97.91 | 104.86 | 104.13 |
| North East and North West | 14.51 | 28.73 | 31.28 | 34.67 | 40.15 | 54.81 | 66.93 | 81.11 | 83.94 | 99.53 |
| North and Central Coast | 20.70 | 34.25 | 39.77 | 44.78 | 50.71 | 61.97 | 82.51 | 99.63 | 107.40 | 107.81 |
| Red river delta | 21.50 | 40.17 | 39.02 | 48.56 | 59.95 | 73.29 | 103.23 | 119.59 | 123.76 | 136.75 |
| South East | 33.15 | 44.14 | 48.18 | 48.14 | 58.68 | 69.46 | 78.59 | 94.84 | 108.13 | 120.70 |
| **Female** |  |  |  |  |  |  |  |  |  |  |
| Central Highlands | 25.68 | 31.43 | 31.71 | 35.22 | 49.69 | 58.79 | 69.95 | 92.61 | 88.27 | 99.43 |
| Mekong river delta | 29.01 | 39.32 | 39.50 | 39.85 | 48.40 | 55.61 | 63.38 | 73.43 | 82.39 | 83.30 |
| North East and North West | 14.51 | 25.63 | 28.63 | 33.11 | 40.15 | 48.80 | 63.16 | 79.52 | 83.94 | 97.72 |
| North and Central Coast | 20.70 | 32.81 | 31.10 | 37.37 | 48.27 | 61.71 | 75.25 | 84.13 | 93.08 | 101.08 |
| Red river delta | 21.50 | 39.46 | 35.77 | 48.56 | 59.95 | 73.29 | 103.23 | 119.59 | 120.12 | 136.75 |
| South East | 22.10 | 36.96 | 38.54 | 43.76 | 51.34 | 63.68 | 73.68 | 91.05 | 104.64 | 110.64 |

Notes: Sample includes households from VHLSS 1992-2016.

**Table A9. Agricultural Wage Rates vs. Minimum Wage Levels**

| Panel A: Ratio of agricultural wage rates to minimum wage rates | | | | | | | | | | |
| --- | --- | --- | --- | --- | --- | --- | --- | --- | --- | --- |
|  | | | | | | | | | | |
|  | 1992 | 1998 | 2002 | 2004 | 2006 | 2008 | 2010 | 2012 | 2014 | 2016 |
| Median |  |  |  |  |  |  |  |  |  |  |
| Male | 0.972 | 1.944 | 1.905 | 1.724 | 1.444 | 1.852 | 2.192 | 1.714 | 1.526 | 1.250 |
| Female | 0.778 | 1.653 | 1.619 | 1.379 | 1.333 | 1.852 | 1.918 | 1.429 | 1.263 | 1.167 |
|  |  |  |  |  |  |  |  |  |  |  |
| Mean |  |  |  |  |  |  |  |  |  |  |
| Male | 0.990 | 1.861 | 1.984 | 1.618 | 1.415 | 1.950 | 1.961 | 1.485 | 1.326 | 1.704 |
| Female | 0.788 | 1.559 | 1.638 | 1.389 | 1.252 | 1.746 | 1.747 | 1.315 | 1.192 | 1.599 |
|  |  |  |  |  |  |  |  |  |  |  |
| Panel B: Percentage of wage rates below the statutory minimum wage | | | | | | | | | | |
|  |  |  |  |  | Agriculture | | | Non-agriculture | | |
|  |  |  |  | 2012 | 2014 | 2016 |  | 2012 | 2014 | 2016 |
| General |  |  |  | 14.53 | 21.62 | 28.43 |  | 6.05 | 8.26 | 11.33 |
| By region | | | |  |  |  |  |  |  |  |
| Central Highlands | | | | 15.81 | 24.41 | 29.82 |  | 4.56 | 6.77 | 8.00 |
| Mekong River Delta | | | | 17.51 | 28.68 | 30.33 |  | 10.91 | 14.01 | 15.68 |
| North and South Central Coast | | | | 16.17 | 22.35 | 29.67 |  | 6.73 | 9.31 | 12.3 |
| North East and North West | | | | 12.07 | 20.8 | 32.16 |  | 4.87 | 5.55 | 9.17 |
| Red River Delta | | | | 25.87 | 23.55 | 28.34 |  | 5.36 | 7.26 | 11.31 |
| South East | | | | 5.49 | 6.98 | 19.74 |  | 4.21 | 6.57 | 9.46 |
| By gender | | | |  |  |  |  |  |  |  |
| Male | | | | 11.51 | 16.88 | 22.87 |  | 4.79 | 6.74 | 9.52 |
| Female | | | | 20.16 | 30.87 | 38.92 |  | 7.83 | 10.32 | 13.7 |

Notes: Monthly minimum wage level applied to domestic firms. In panel A, monthly minimum wage is converted to daily minimum wage using the median (mean) working days per month of agriculture workers in the sample (VHLSS). In panel B, monthly minimum wage is converted to daily rate using the median working days per month of agriculture/non-agriculture workers in the sample (LFS), which includes all workers aged 15-60 (excluding those who receive wage in kind or are unpaid).

**Table A10. Regression results on land productivity of spring ordinary rice**

|  | **1992/98** | **2006/08** | **2010/12** | **2014/16** |  |
| --- | --- | --- | --- | --- | --- |
|  | **(1)** | **(2)** | **(3)** | **(4)** | **(5)=(4)-(1)** |
| Log total area of spring ordinary rice | -0.1259*** | -0.0426*** | -0.0464*** | -0.0331** | 0.0544** |
|  | (0.0214) | (0.0126) | (0.0142) | (0.0147) | (0.0228) |
| Male household head | 0.0062 | 0.0044 | -0.0568 | 0.0262 | 0.0235 |
|  | (0.0366) | (0.0342) | (0.0452) | (0.0454) | (0.0514) |
| Age of household head | -0.0005 | -0.0006 | 0.0009 | 0.0004 | 0.0010 |
|  | (0.0012) | (0.0009) | (0.0017) | (0.0013) | (0.0016) |
| Highest education of household members | 0.0050 | -0.0021 | -0.0008 | 0.0045 | -0.0041 |
|  | (0.0056) | (0.0052) | (0.0040) | (0.0049) | (0.0055) |
| Number of male members | 0.0070 | 0.0015 | -0.0111 | 0.0104 | 0.0132 |
|  | (0.0177) | (0.0133) | (0.0168) | (0.0120) | (0.0188) |
| Household size | 0.0010 | -0.0010 | 0.0081 | -0.0066 | -0.0123 |
|  | (0.0102) | (0.0086) | (0.0080) | (0.0081) | (0.0109) |
| Year | 0.0385*** | -0.0046 | 0.0349*** | -0.0056 | -0.0270*** |
|  | (0.0029) | (0.0034) | (0.0035) | (0.0040) | (0.0037) |
| Observations | 4932 | 3465 | 3247 | 2970 |  |
| R-squared | 0.229 | 0.010 | 0.111 | 0.010 |  |

Notes: Notes: Sample includes households from VHLSS 1992-2016. Standard errors in parentheses, clustered at the commune level. The variables "Log total area of spring ordinary rice" centered around their sample means. * p<0.10, ** p<0.05, *** p<0.01.

**Table A11. Regression results on land productivity of autumn ordinary rice**

|  | **1992/98** | **2006/08** | **2010/12** | **2014/16** |  |
| --- | --- | --- | --- | --- | --- |
|  | **(1)** | **(2)** | **(3)** | **(4)** | **(5)=(4)-(1)** |
| Log total area of autumn ordinary rice | -0.1995*** | -0.0851*** | -0.0250 | -0.0152 | 0.0710* |
|  | (0.0360) | (0.0197) | (0.0255) | (0.0248) | (0.0383) |
| Male household head | -0.0669 | 0.0369 | 0.0354 | 0.0044 | 0.0023 |
|  | (0.0527) | (0.0599) | (0.0578) | (0.0354) | (0.0504) |
| Age of household head | -0.0006 | 0.0009 | 0.0043 | 0.0014 | 0.0012 |
|  | (0.0021) | (0.0018) | (0.0035) | (0.0021) | (0.0022) |
| Highest years of schooling of household members | 0.0153** | -0.0034 | 0.0014 | 0.0019 | -0.0115** |
|  | (0.0063) | (0.0062) | (0.0058) | (0.0037) | (0.0056) |
| Number of male members | 0.0300 | -0.0164 | 0.0332 | 0.0300** | 0.0089 |
|  | (0.0223) | (0.0181) | (0.0250) | (0.0135) | (0.0205) |
| Household size | 0.0117 | 0.0167* | -0.0090 | -0.0128 | -0.0236** |
|  | (0.0124) | (0.0101) | (0.0142) | (0.0078) | (0.0116) |
| Year | 0.0229*** | 0.0301*** | 0.0291*** | 0.0090** | -0.0294*** |
|  | (0.0050) | (0.0056) | (0.0059) | (0.0036) | (0.0044) |
| Observations | 4431 | 2728 | 2514 | 2463 |  |
| R-squared | 0.107 | 0.054 | 0.057 | 0.013 |  |

Notes: Sample includes households from VHLSS 1992-2016. Standard errors in parentheses, clustered at commune level. The variables "Log total area of autumn ordinary rice" centered around their sample means. * p<0.10, ** p<0.05, *** p<0.01.

**Table A12. Mean share of agricultural output (by value)**

|  | Total farms | | | Small farms | | | Big farms | | |
| --- | --- | --- | --- | --- | --- | --- | --- | --- | --- |
|  | 2002 | 2010 | 2016 | 2002 | 2010 | 2016 | 2002 | 2010 | 2016 |
| Rice | 0.408 | 0.507 | 0.368 | 0.401 | 0.554 | 0.428 | 0.377 | 0.379 | 0.300 |
| Ordinary rice | 0.385 | 0.475 | 0.340 | 0.384 | 0.523 | 0.411 | 0.357 | 0.357 | 0.268 |
| Sticky rice | 0.017 | 0.019 | 0.016 | 0.011 | 0.014 | 0.004 | 0.018 | 0.019 | 0.032 |
| High-quality rice | 0.006 | 0.013 | 0.013 | 0.005 | 0.018 | 0.014 | 0.002 | 0.003 | 0.000 |
| Other food crops | 0.100 | 0.110 | 0.123 | 0.083 | 0.092 | 0.104 | 0.132 | 0.151 | 0.157 |
| Industrial crops | 0.095 | 0.100 | 0.126 | 0.050 | 0.041 | 0.044 | 0.191 | 0.236 | 0.278 |
| Fruits | 0.068 | 0.051 | 0.062 | 0.074 | 0.048 | 0.054 | 0.068 | 0.054 | 0.062 |
| Aquaculture | 0.054 | 0.032 | 0.037 | 0.050 | 0.027 | 0.032 | 0.077 | 0.045 | 0.045 |
| Livestock | 0.274 | 0.199 | 0.284 | 0.343 | 0.238 | 0.338 | 0.156 | 0.134 | 0.158 |

Notes: Sample includes households from VHLSS 2002, 2010, and 2016. Industrial crops include both annual and perennial crops. Other food crops include vegetables and other staples. Small (big) farms are those whose cultivated land (annual crop land, perennial crop land, and water surface) falls within the bottom (top) 20%.

**Table A13. Share of food expenditures auto-consumed by farming households**

|  | 1992 | 1998 | 2010 | 2012 | 2014 | 2016 |
| --- | --- | --- | --- | --- | --- | --- |
| Mean | 0.507 | 0.471 | 0.269 | 0.265 | 0.245 | 0.235 |
| S.D | 0.255 | 0.224 | 0.203 | 0.221 | 0.216 | 0.210 |
| Median | 0.535 | 0.504 | 0.249 | 0.240 | 0.212 | 0.197 |

Notes: Sample includes households from VHLSS 1992-2016.

**Table A14. Regression results of per capita consumption expenditure on education and landholdings**

|  | 2004 | 2006 | 2008 | 2012 | 2014 | 2016 |
| --- | --- | --- | --- | --- | --- | --- |
|  | (1) | (2) | (3) | (4) | (5) | (6) |
| Log of total land owned (square meters) | 0.1299*** | 0.1272*** | 0.1315*** | 0.1455*** | 0.1159*** | 0.1311*** |
|  | (0.0053) | (0.0051) | (0.0084) | (0.0050) | (0.0061) | (0.0052) |
| Male household head | 0.0568*** | 0.0534*** | 0.0145 | 0.0541*** | 0.0795*** | 0.0625*** |
|  | (0.0103) | (0.0140) | (0.0138) | (0.0111) | (0.0180) | (0.0119) |
| Age of household head | -0.0017*** | -0.0012*** | -0.0007* | -0.0013*** | -0.0003 | -0.0007** |
|  | (0.0003) | (0.0004) | (0.0004) | (0.0003) | (0.0005) | (0.0003) |
| Highest grade completed of household members | -0.0180*** | -0.0123*** | -0.0161*** | -0.0101*** | -0.0126*** | -0.0104*** |
|  | (0.0015) | (0.0025) | (0.0022) | (0.0015) | (0.0023) | (0.0018) |
| Number of male members | -0.0075 | -0.0202** | -0.0057 | -0.0126** | -0.0107 | -0.0131** |
|  | (0.0049) | (0.0102) | (0.0071) | (0.0060) | (0.0083) | (0.0062) |
| household size | -0.0056 | -0.0066 | -0.0177*** | -0.0125*** | -0.0093 | -0.0140*** |
|  | (0.0035) | (0.0043) | (0.0042) | (0.0041) | (0.0057) | (0.0041) |
| Observations | 4899 | 4638 | 4455 | 4815 | 2623 | 4626 |
| R-squared | 0.236 | 0.137 | 0.173 | 0.352 | 0.246 | 0.313 |

Notes: The sample includes households from VHLSS 1992-2016. Standard errors in parentheses, clustered at the commune level. Regional dummies are included in all regressions. * p<0.10, ** p<0.05, *** p<0.01

**Table A15. Regression results of per capita consumption expenditure on education and landholdings**

|  | 2004 | 2006 | 2008 | 2012 | 2014 | 2016 |
| --- | --- | --- | --- | --- | --- | --- |
|  | (1) | (2) | (3) | (4) | (5) | (6) |
| Log of total land owned (square meters) | -0.1009*** | -0.0898*** | -0.1537*** | -0.1108*** | -0.0936*** | -0.1050*** |
|  | (0.0053) | (0.0053) | (0.0250) | (0.0055) | (0.0061) | (0.0056) |
| Male household head | -0.0856*** | -0.0737*** | -0.0024 | -0.0635*** | -0.0778*** | -0.0706*** |
|  | (0.0119) | (0.0150) | (0.0655) | (0.0138) | (0.0184) | (0.0147) |
| Age of household head | 0.0031*** | 0.0025*** | -0.0071*** | 0.0033*** | 0.0012** | 0.0020*** |
|  | (0.0003) | (0.0004) | (0.0018) | (0.0004) | (0.0005) | (0.0004) |
| Highest grade completed of household members | 0.0117*** | 0.0061** | 0.0285** | 0.0019 | 0.0023 | 0.0014 |
|  | (0.0016) | (0.0025) | (0.0123) | (0.0017) | (0.0023) | (0.0020) |
| Number of male members | 0.0147*** | 0.0182* | -0.0045 | 0.0042 | 0.0073 | 0.0120 |
|  | (0.0053) | (0.0103) | (0.0256) | (0.0069) | (0.0091) | (0.0074) |
| household size | -0.0050 | -0.0032 | 0.0196 | 0.0058 | 0.0043 | 0.0085* |
|  | (0.0037) | (0.0045) | (0.0208) | (0.0048) | (0.0059) | (0.0048) |
| Observations | 4899 | 4638 | 4455 | 4815 | 2623 | 4626 |
| R-squared | 0.157 | 0.080 | 0.014 | 0.216 | 0.164 | 0.173 |

Notes: The sample includes households from VHLSS 1992-2016. Standard errors in parentheses, clustered at the commune level. Regional dummies are included in all regressions. * p<0.10, ** p<0.05, *** p<0.01

**Table A16.** **Regression results of per capita consumption expenditure on education and landholdings**

|  | 1992 | 2004 | 2006 | 2008 | 2012 | 2014 | 2016 |
| --- | --- | --- | --- | --- | --- | --- | --- |
|  | (1) | (2) | (3) | (4) | (5) | (6) | (7) |
| Log of total land owned (square meters) | 0.0638*** | 0.0685*** | 0.0490*** | 0.0585*** | 0.0305*** | 0.0348*** | 0.0154 |
|  | (0.0171) | (0.0080) | (0.0079) | (0.0076) | (0.0090) | (0.0103) | (0.0098) |
| Male household head | 0.0010 | 0.0322* | 0.0361** | 0.0212 | 0.0353 | 0.0414 | 0.0446* |
|  | (0.0225) | (0.0173) | (0.0181) | (0.0185) | (0.0214) | (0.0302) | (0.0237) |
| Age of household head | 0.0067*** | 0.0041*** | 0.0037*** | 0.0030*** | 0.0020*** | 0.0045*** | 0.0039*** |
|  | (0.0007) | (0.0005) | (0.0005) | (0.0005) | (0.0006) | (0.0008) | (0.0006) |
| Highest grade completed of household members | 0.0513*** | 0.0674*** | 0.0694*** | 0.0645*** | 0.0774*** | 0.0753*** | 0.0736*** |
|  | (0.0058) | (0.0025) | (0.0025) | (0.0028) | (0.0028) | (0.0038) | (0.0037) |
| Number of male members | 0.0120 | 0.0003 | 0.0118 | 0.0178** | 0.0267** | 0.0064 | 0.0009 |
|  | (0.0077) | (0.0073) | (0.0076) | (0.0089) | (0.0107) | (0.0139) | (0.0111) |
| household size | -0.0802*** | -0.1208*** | -0.1228*** | -0.1458*** | -0.1424*** | -0.1560*** | -0.1651*** |
|  | (0.0061) | (0.0055) | (0.0054) | (0.0063) | (0.0077) | (0.0094) | (0.0081) |
| Observations | 3513 | 4899 | 4638 | 4455 | 4815 | 2623 | 4626 |
| R-squared | 0.266 | 0.357 | 0.349 | 0.333 | 0.323 | 0.384 | 0.366 |

Notes: The sample includes households from VHLSS 1992-2016. Standard errors in parentheses, clustered at the commune level. Regional dummies are included in all regressions. * p<0.10, ** p<0.05, *** p<0.01
